# Acknowledgement to Reviewers of *Molecules* in 2014

**DOI:** 10.3390/molecules20010693

**Published:** 2015-01-07

**Authors:** Molecules Editorial Office

**Affiliations:** MDPI AG, Klybeckstrasse 64, CH-4057 Basel, Switzerland

The editors of *Molecules* would like to express their sincere gratitude to the following reviewers for assessing manuscripts in 2014:

Abashev, G. G.
Abass, Khaled
Abbate, Sergio
Abbehausen, Camilla
Abdelmohsen, Usama Ramadan
Abdullayev, Elshad
Abe, Koichi
Abe, Naohito
Abe, Ryu
Abel, S.
Åberg, Ola
Abliz, Zeper
Acero, Nuria
Achelle, Sylvain
Adam, Rosa
Adam, Vojtech
Adams, Joshua
Adams, Michael
Adams, Timothy E.
Adeloye, Adewale O.
Adriano, Marocco
Afarinkia, Kamyar
Afiyatullov, Shamil Sh.
Afonso, Carlos A.M.
Agamennone, Mariangela
Ågren, H.
Agrios, Alexander G.
Agrofoglio, Luigi A
Aguilar, Cristóbal N.
Aguirre, Pedro
Ahn, Joong-Hoon
Ahn, Kyo Han
Ahn, Soon-Cheol
Ahrends, Robert
Aidoudi, Farida H.
Ainslie, Kristy M.
Aires, Alfredo
Ajikumar, Parayil Kumaran
Akagawa, Mitsugu
Akai, Shuji
Akira, Kazuki
Alabugin, Igor V.
Alam, Mohammad A.
Alam, M S.
Alami, Jones
Alavez, Silvestre
ALBERICIO, Fernando
Albert, Nick
Albini, Angelo
Albrectsen, Benedicte R.
Albuquerque, Magaly
Alcântara, Ana C. S.
Alcaraz, Gilles
Aldabbagh, Fawaz
Aldrich-Wright, Janice R.
Alexopoulos, A.
Alezra, Valérie
Algar, W. Russ
Ali, Shaikh A.
Ali, Simi
Alisi, Anna
Allcock, Harry R.
Allemann, Rudolf K.
Allen, N.S.
Allen, William J.
Allgaier, Jürgen
Almajano, María Pilar
Al-Masum, Mohammad
Almeida, Wanda
Almerico, Anna Maria
Almqvist, Fredrik
Al-Musayeib, Nawal M.
Al-Obaidi, Hisham
Alonsoa, Francisco
Al-Othman, Zeid
Altman, Ryan A.
Alvarado, Vladimir
Alvarez-Builla, Julio
Alvarez-Lorenzo, Carmen
Alvarez-Suarez, José Miguel
Alves, Gilberto
Alves, Isabel D.
Alves, Maria José
Amadelli, R.
Amarasekara, Ananda S.
Ambudkar, Suresh V.
Amić, Dragan
Amo-Ochoa, Pilar
Ananikov, Valentine P.
Anastyuk, Stanislav D.
Anderson, Marilyn
Andjelkovic, Anuska V.
Andrade, Carolina Horta
Andrade, Leandro
Andrade, Paula Andrade
Andrade, Suzana M.
Andrade-Cetto, Adolfo
Andreoli, Roberta
Andres, Juan
Andrushchenko, Valery
Andújar, Isabel
Angela, Bisio
Angulo, Javier
Annaert, Pieter
Anraku, Makoto
Antelmann, Haike
Anthony, Okoh I
Antonchick, Andrey
Anwar, Sri Haryani
Aoyagi, Noboru
Apak, Reşat
Apte, Suneel S.
Arai, Masayoshi
Arai, Takayoshi
Arapitsas, Panagiotis
Araújo, Marcelo Gonzaga de Freitas
Araujo, Maria T. de
Arbault, Stephane
Arbona, Vicent
Arce-Estrada, Elsa M.
Arceusz, Agnieszka
Archibald, Steve J.
Arena, C.
Arias-Pérez, María-Selma
Ariga, Hiroyoshi
Ariga, Katsuhiko
Arion, Vladimir
Ariyama, Kaoru
Armentano, Donatella
Armishaw, Christopher J.
Arnason, John T.
Arnson, Yoav
Arrabal, Carlos
Arranz, Elena
Arrieta, Jesús
Arsu, Nergis
Artese, Anna
Aruoma, Okezie
Asai, Teigo
Asakawa, Yoshinori
Asano, Keisuke
Ashton, Kevin
Assadi, Aymen Amine
Assfeld, Xavier
Ata, Athar
Atanasov, Atanas G.
Athayde, Margareth Linde
Atkinson, Ross G.
Attoub, Samir
Aubé, Jeffrey
Augugliaro, Vincenzo
Avarvari, Narcis
Awale, Suresh
Awual, M. Rabiul
Azémaa, Joëlle
Azuma, Akifumi
Bach, Horacio
Bacher, Adelbert
Bácskay, Ildikó
Baczek, Tomasz
Badawi, Michael
Badinga, Lokenga
Baek, Nam In
Bagla, Victor
Bai, Gang
Bai, Xiaochun
Bailey, David
Bailly, Christian
Bakare, Oladapo
Balaz, Milan
Bald, Ilko
Balemba, Onesmo B.
Ball, Zachary T.
Baltina, L. A.
Balzarini, Jan
Bana, Emilie
Banerjee, Sanjeev
Banfi, Luca
Bangoura, Berit
Banister, Samuel
Bankova, Vassya
Bannwarth, Markus B.
Bao, Jin-Ku
Barakate, Abdellah
Baraldi, Pier Giovanni
Baranac-Stojanovi, Marija
Barbayianni, Efrosini
Barbera, Mariagnese
Barbosa, Luiz C.A.
Barceló, Francisca
Barea, Carlos
Barker, David
Barkley, Noelle
Barolo, Claudia
Baron, V T
Barrajón-Catalán, Enrique
Barreira, João
Barreiro, Eliezer Jesús
Barrena, Esther
Barreto, Maria Do Carmo
Barrett, Ann
Barrio, Pablo
Barros, Lillian
Barth, Brian M.
Basak, Saroj K.
Basha, Riyaz
Basini, Giuseppina
Bassyouni, Fatma A.
Batalla, Pilar
Batchelor, Warren
Bates, Philip D.
Battino, Maurizio
Baxendale, Ian
Bayindir, Zerrin Sezgin
Baykal, A.
Baziard, Geneviève
Bazylak, Grzegorz
Bazylińska, Urszula
Bazylko, Agnieszka
Bazzocchi, Isabel L.
Beauregard, Marc
Beck, Stephanie
Beckmann, Manfred
Bednarczyk-Cwynar, Barbara
Bednarski, Patrick
Beeren, Sophie
Beerhues, Ludger
Begum, Shamim A.
Bekhit, Aladin
Beletskaya, Irina P.
Belfield, Kevin
Belfort, Georges
Bell, Stephen
Bello, Nicholas T.
Belov, Georgiy
Bełtowski, Jerzy
Ben-Arie, Ruth
Benavides, Jorge
Benbrook, Doris Mangiaracina
Benelli, Giovanni
Benes, Petr
Benhida, Rachid
Béni, Szabolcs
Benini, Stefano
Benoist, Eric
Benoist, Hervé
Beránek, Radim
Berenguer-Murcia, Ángel
Bergheim, Ina
Beringhs, André O’Reilly
Bernardo-Gil, M. Gabriela
Bernini, Roberta
Bernot, Randall
Berruga, Maria Isabel
Berry, John
Berthod, Alain
Bertini, Luca
Berton, Paula
Besada, Cristina
Bescond, J.
Besson, Thierry
Bester, Megan J.
Beutler, John A.
Bhadani, Avinash
Bhagavathy, Ganga
Bhowmik, Pradip
Biagini, Giuseppe
BIAN, Zhaoxiang
Bianchi, Antonio
Biasutto, Lucia
Bielawski, Christopher W.
Biesaga, Magdalena
Bijak, Michał
Billard, Thierry
Bin, Fu
Biot, Christophe
Birch, Edward
Birkedal, V.
Birney, David M.
Bishop-Hurley, Sharon
Bizukojc, Marcin
Bizzarri, Anna Rita
Blade Segara, Maria Cinta
Blanchard, Scott C.
Blanco, Rosa M
Blandino, G
Blank, Kerstin
Bledzki, Andrzej K.
Bocchinfuso, Gianfranco
Boechat, Nubia
Boess, Frank G.
Bofill, Josep Maria
Bogár, Krisztián
Bogel-Łukasik, Rafal
Böhm, Volker
Boido, Vito
Boligon, Aline Augusti
Bolm, Carsten
Bonaccorsi, Ivana
Bondar, Mykhailo V.
Bonnet, Sylvestre
Bontemps, Nataly
Booth, Stephanie A.
Boparai, Hardiljeet
Boratynski, Janusz
Boratyński, Przemysław
Borges, Anabela
Borges, João P.M.R.
Boros, Eszter
Borowska, Marta
Borrego, Eli
Bouajila, Jalloul
Boué, Steve
Bouquillon, Sandrine
Bourgault, Steve
Bourgeois, Dominique
Bou-Saab, Hamid
Bousbaa, Hassan
Boylan, Fabio
Bradshaw, Tracey D.
Braeuer, Andreas
Braga, Valdir A.
Braissant, O.
Brancale, Andrea
Brandão, Maria das Graças
Bratkovic, Tomaz
Bratsos, Ioannis
Braun, Ruedi K.
Bravo, Laura
Bravo-Díaz, Carlos
Brazzelli., V
Brecker, Lothar
Breeman, Wouter A.P.
Breinbauer, Rolf
Breitenfeld Granadeiro, Luiza
Bremner, John B.
Brenna, Elisabetta
Brenndörfer, Erwin
Brigante, Marcello
Brobeil, Alexander
Broberg, Anders
Brock, Matthias
Broderick, Patricia A.
Bromann, Kirsi
Bromke, Mariusz A.
Bronze, Maria
Brooke, Basil
Brown, Alan B.
Brown, Alex
Brown, J. E.
Brown, Lindsay
Browne, Wesley R.
Bruce, Duncan
Bruckner, Christian
Brudzynski, Katrina
Brun, Reto
Brunetti, Cecilia
Brüning, Ansgar
Bruno, Maurizio
Brycki, Bogumił
Bu, Xianzhang
Buffon, Andréia
Bukowska, Bożena
Bulgariu, Laura
Buolamwini, John K.
Burketova, L
Burkholz, Torsten
Buron, Frédéric
Burritt, David
Burt, Sara A.
Buszewski, BogusÅ‚aw
Bystrowska, Beata
Cabaleiro, Noelia
Caboni, Pierluigi
Cabrera, Margarita Gutiérrez
Cadierno, Victorio
Cahill, Paul
Cai, Jianfeng
Cai, Kaiyong
Cai, Wenqing
Cai, Xiang-hai
Cai, Yang
Calani, Luca
Calderon, Angela
Calderone, Richard
Calderone, Vincenzo
Calero, Nuria
Calhelha, Ricardo C.
Caliandro, Rocco
Caliceti, Paolo
Caliendo, Giuseppe
Calin, George A.
Calín-Sánchez, Ángel
Calla-Magariños, J.
Callery, Patrick S.
Calligaris, Sonia
Calogeropoulou, Theodora
Calokerinos, Anthony
Camacho, M. Encarnación
Camara, Bilal
Câmara, José
Camerel, Franck
Cameron, Luiz-claudio
Campagna, Francesco
Campagne, Jean-Marc
Campiglia, Pietro
Campillo, Nuria E.
Campos, Patrícia M. B. G. Maia
Canas, Sara
Cantí, Carles
Cao, Shugeng
Cao, Wei
Cao, Xueli
Capasso, Andrea
Carbone, Anna
Carbone, K.
Carbonell, Pablo
Carbonell-Bejerano, Pablo
Cardeno, Ana
Cardona, Wilson
Cardoso, Susana M.
Carell, Thomas
Caretto, Sofia
Carloni, Paolo
Carlsson, Uno
Carluccio, Maria Annunziata
Carnero, Amancio
Caro, Andres A.
Carpene, Christian
Carradori, Simone
Carraher Jr., Charles E.
Carrasco, Cristina
Carrier, Danielle Julie
Carro-Díaz, A.m.
Carrubba, Alessandra
Carta, Antonio
Carta, Fabrizio
Carvalho, Luiz Joaquim Castelo Branco
Casas, Maria Isabel
Casassa, Luis Federico
Cascio, Michael
Casella, Luigi
Caselli, Alessandro
Castaneda, Homero
Castellari, Massimo
Castilho, Marcelo
Castrén, Maija L.
Castro, Angeles
Castro, Eduardo
Catalá, Myriam
Caulton, Kenneth
Cavada, Benildo
Cavalcanti, Bruno
Cavalli, Roberta
Cavar, Sanja
Cebolla, Vicente L.
Celia, Christian
Cellini, Francesco
Cenac, Nicolas
Cenci, Elio
Cerecetto, Hugo
Cermak, R.
Černík, Miroslav
Cerra, M.c.
Ceruso, Mariangela
Cesare, Angela Di
Céspedes, Carlos
Cha, Hyukjin
Chachua, Tamar
Chae, Han-Jung
Chai, Han-Ha
Chai, Yifeng
Chaimbault, Patrick
Champagne, Benoît
Chamulitrat, Walee
Chan, Shun-Wan
Chan, Thomas
Chandra, Dhyan
Chang, Chang-Tang
Chang, Cicero L. T.
Chang, Fang-Rong
Chang, Gwo-Jyh
Chang, Hsieh
Chang, Hsueh-Wei
Chang, Il-moo
Chang, Long-Sen
Chang, Rakwoo
Chang, Shang-Tzen
Chang, Wei-chiao
Chang, Woo-Suk
Chang, Yuan-Shiun
Chang, Yung-Ho
Chaniotakis, Nikos
Chatre, Laurent
Chaves, Mariana H.
Chaves, Susana Rodrigues
Chavez, Ferman
Che, Chun-Tao
Chemat, Farid
Chen, Banglin
Chen, Bo
Chen, Chao
Chen, Chi
Chen, Chiung-Mei
Chen, Chung-Yi
Chen, Feng
Chen, Fenghua
Chen, Fusheng
Chen, Haifeng
Chen, Hao
Chen, Hong-Li
Chen, Hubiao
Chen, Huixiong
Chen, Jih-Jung
Chen, Jyh-Ping
Chen, Kew-Yu
Chen, Kuan-chou
Chen, Lanming
Chen, Ligong
Chen, Lih-Geeng
Chen, Pai-Shan
Chen, Pin-Shern
Chen, Qiao-Hong
Chen, Qi-He
Chen, Qing-Xi
Chen, Shao-Yu
Chen, Shih-Yuan
Chen, Wansheng
Chen, Wei-Jen
Chen, Wen Yong
Chen, Wen-bin
Chen, Xi
Chen, Xiao-Qing
Chen, Xiaoyan
Chen, Xin
Chen, Xiuping
Chen, Yen-Chou
Chen, Ying
Chen, Yongli
Chen, Yu
Chen, Yunfeng
Chen, Zhonghua
Cheng Lian Ee, Gwendoline
Cheng, Heung-Chin
Cheng, Juei-tang
Cheng, Kuan-chen
Cheng, Po-Ching
Cheng, Ran
Cheng, Tak
Cheng, Xinlai
Cheng, Yongxian
Cheng, Zhi-Hui
Cherng, Jong-yuh
Cherniack, E. Paul
Cheung, Randy Chi Fai
Chevalot, Isabelle
Chew, Eng-Hui
Chi, Ki-Whan
Chi, Robin Yonggui
Chia, Win-Long
Chiacchio, Ugo
Chianese, Giuseppina
Chiang, Po-Chang
Chiang, Shu-Hua
Chiantore, Oscar
Chiashi, Shohei
Chieli, Elisabetta
Chien, Shih-Chang
Chilin, Adriana
Chiocchia, Gilles
Chiolerio, Alessandro
Chiou, Hui-ling
Chiou, Shih-hwa
Chiriano, Gianpaolo
Chirumbolo, Salvatore
Chiu, Chien-Chih
Chiu, Tsai-Hsin
Cho, Chang-Won
Cho, Hoon
Cho, Man-Ho
Cho, Sung Yun
Cho, Sunghee
Cho, William
Chobot, Vladimir
Choi, Chang-Hwan
Choi, Hyeok
Choi, Hyoung Jin
Choi, Hyung-kyoon
Choi, Je-Min
Choi, Sanghoon
Choi, Yongseok
Choi, Young Hae
Choi, Yung-Hyun
Cholewinski, Grzegorz
Chong, Youhoon
Chorianopoulos, Nikos G.
Chou, Chang-hung
Chou, Tsui-Fen
Chow, Moses Sing Sum
Chrissanthopoulos, Athanassios
Christov, Christo Z.
Chrobok, A.
Chrzumnicka, E.
Chu, Fang-hua
Chu, Ivan K.
Chu, Qianli
Chuang, John Shih-ching
Chuang, Ta-hsien
Chuburu, Françoise
Chui, Wai Keung
Chun, Wanjoo
Chung, Hoi Sung
Chyau, Charng-Cherng
Ciappellano, Salvatore
Cicero, Arrigo F.G.
Cichero, Elena
Cidade, Honorina
Cieśla, Łukasz
Církva, Vladimír
Citterio, Barbara
Ciuffi, Kátia J.
Clark, Lindsay V.
Claudia, Grothe, P
Clericuzio, Marco
Coasne, Benoit
Coates, Christopher J
Cobb, Alexander
Cock, Ian
Coelho, Elisabete
Coelho, Jorge F.
Coelho, José A
Coelho, Paulo
Coiffard, Laurence J M
Coimbra, Eliane Fields
Colacino, Joseph M.
Coles, Gerald
Coley, Helen M.
Coll, Josep
Collier, C Patrick
Collina, Simona
Collins, Maurice
Colombo, Maria Laura
Colorado, Jhonny
Colotti, Gianni
Coman, Gigi
Combet, Emilie
Concannon, Caoimhin
Conde, José
Condorelli, Momina
Conti, Barbara
Cooper, Arthur
Corbi, Pedro P.
Cordoba-Diaz, D.
Corke, Harold
Corradini, Danilo
Correia Soeiro, Maria de Nazaré
Corsi, Fabio
Cosentino, Marco
Costa, Paulo J
Costantini, Susan
Costello, Derek A.
Coussot, P.
Cozzi, Renata
Cozzolino, Daniel
Crane, Edward J.
Crawford, Philip W.
Creczynski-Pasa, Tania
Crespo, Margarita
Cristobal Lopez, J.
Crupi, Pasquale
Csámpai, Antal
Csuk, René
Cubilla-Rios, Luis
Cubry, Philippe
Cuca Suárez, Luis Enrique
Cuendet, Muriel
Culf, Adrian S.
Cuny, Gregory D.
Curtis, Jonathan M.
Custódio, Luísa
Cvengroš, Ján
Cycoń, Mariusz
Czarnocki, Zbigniew
D’ischia, Marco
Da Costa, Fernando B.
da Silva, A. Moreira
Da Silva, Albérico
Da Silva, Elisiário Tavares
da Silva, Gustavo
da Silva, M. Fátima das G.F.
Dabek-Zlotorzynska, Ewa
D'abrosca, Brigida
Dai, Jungui
Daiber, Andreas
Daly, Norelle
D'Ambrosio, Michele
Dandapani, Sivaraman
Dangles, Olivier
Daniel, Kenyon
Darvesh, Sultan
Darvin, M.E.
Das, Kalyan
David Wang, Hui-Min
David, Jorge
Davies, Mike
Davis, Matthew C.
De Albuquerque, Julianna F.C.
De Almeida Coelho De Sousa, Maria João
de Assis, Adriano M.
De Diego, Marta
De Falco, Enrica
de Feo, Vincenzo
De Jesús Ornelas-Paz, José
de Jonge, Ronnie
De Kimpe, Norbert
de Kock, Carmen
De La Fuente, M.A.
De La Herrán, Roberto
de la Hoz, Antonio
De la Mata, F. Javier
De la Monte, Suzanne
de la Torre, Rafael
De La Vega, José Manuel García
De Leon, Marino
De Marino, Simona
de Medina, F Sánchez
De Oliveira, Anderson R. M.
de Pascual-Teresa, Sonia
De Spirt, Silke
De Tommasi, Nunziatina
De Visser, S.p.
De Vita, Daniela
de Vita, Pasquale
De Wild, Paul. J.
Dealtry, Gill
DeBolt, Seth
Debyser, Zeger
Dechoux, Luc
Deconinck, Eric
Decréau, Richard
Decuypere, Jean-Paul
Decuzzi, Paolo
Dedyukhina, Emiliya G.
Defrancq, Eric
Deharo, Eric
Deivanayagam, Champion
Delgado Olguín, Paul
Delgado, Alejandra
Delgado, Angel
Della Ca, Nicola
Delmastro-Greenwood, Meghan
Dembinski, Roman
Demchenko, Alexei V.
Demetrescu, I.
Demidova-Rice, Tatiana
Demopoulos, Vassilis J.
Dénès, Fabrice
Denev, Petko
Deng, Lih-Wen
Deng, Lisheng
Dennison, Sarah
Denny, Bill
Denti, Michela A.
Derbré, Séverine
Dernovics, Mihály
D'Errico, Stefano
Desai, Dhimant
Desbois, Andrew
Desbois-Mouthon, Christèle
Desobry, Stéphane
Destaillats, Hugo
Destandau, Emilie
Devred, F.
Dharmarajan, Arunasalam
Dhawan, Deepika
Dhossche, Dirk
Di Donnia, Leonardo Di Donna
Di Santo, Roberto
Di Venere, Donato
Diana, Marina
Diana, Patrizia
Diao, GuoWang
Diaz, Isabel
Díaz, Isabel
Diaz-Fernandez, Yuri
Díaz-Moreno, Amanda Consuelo
Díaz-Ricci, Juan C.
Dietrich, Gilles
Diez, David
Dijkwel, Paul
Dimitroff, Charles J.
Dimmock, Jonathan R.
Dinadayalane, Tandabany C.
Ding, Bao-Jian
Ding, Gang
Ding, Jiandong
Ding, Ye
Ding, Yi
Ding, Yuqiang
Ding, Zhong-Tao
Dinjaski, Nina
Djoko, Karrera Y.
Długońska, Henryka
Dobrikov, Georgi
Docheva, Denitsa
Doležal, K.
Domcke, Wolfgang
Domingues, M. Rosário M.
Dominguez, Herminia
Dominguez-Perles, Raul
Dominguez-Ramos, Antonio
Dong, Mingsheng
Dong, Tina T. X.
Donno, D.
Dontas, Ismene A.
D'orazio, John
Dory, Yves L.
dos Santos, Jean Leandro
Doubleday, Charles
Dowling, Kim
Downer, Eric
Downey, Gerard
Pessoa, Otilia D.L.
Drager, Luciano F.
Dragutan, Valerian
Drašar, Pavel
Drbohlavova, Jana
Dreymüller, Daniela
Drogoudi, Pavlina D.
Drummen, Gregor
Du, Yuguo
Du, Yunfei
Duan, Jin-Ao
Duan, Jinyou
Duan, Kow-jen
Duan, Xiaohui
Duarte, Ana Paula
Dudley, Ed
Dudziak, Diana
Duffy, C. D. P.
Duh, Pin-Der
Dumont, Matthieu
Duncan, James
Durackova, Z.
Durán, Nelson
Durand, Thierry
Durrieu, V.
Dutschk, Victoria
Dutta, Sujoy
Dziedzic, Arkadiusz
Easmon, Johnny
Ebeler, Susan E.
Ebrahimi, Azadeh
Ebrahimi, Samad
Ebrahimzadeh, M.A.
Echevarria, Aurea
Echeverri, Fernando
Ecker, Gerhard F.
Eckert, Gunter P.
Edkins, Adrienne L.
Eduardo-Figueira, Maria
Ee, Pui-Lai Rachel
Egerton, T.A.
Eglin, David
Eiroa, José Luis
Ekuni, Daisuke
El Kaïm, Laurent
El-Achkar, Tarek
Elaissari, Abdelhamid
El-Aneed, Anas
Elhissi, Abdelbary
Eliyan, Faysal Fayez
El-Kareh, Ardith W.
Elkhayat, Ehab S.
Elling, Lothar
Elliot, Carole
Eloff, Jacob
Van Damme, Els J.M.
El-Sayed, Wael A.
El-Seedi, Hesham R.
Emery, Flávio
Enchev, Venelin
Enderlein, Jörg
Engel, Matthias
Enriquez, Rosario
Eparvier, Véronique
Epting, Conrad L.
Erb, U.
Erben, Mauricio F.
Ermakova, Svetlana P.
Erxleben, Andrea
Escuredo, Olga
Espinosa-Aguirre, Jesús Javier
Esposito, Emilio Xavier
Esposito, G.
Esposito, Sergio
Esteban, Maria Ángeles
Esteve-Romero, Josep
Estevinho, Leticia
Estrada-Reyes, Rosa
Estrada-Soto, Samuel
Esumi, Tomoyuki
Evidente, Antonio
Fabian, Walter M.F.
Fabio, Giovanni Di
Fabroni, Simona
Failla, Mark L.
Fairlamb, Ian
Faleiro, Maria L.
Falodun, A
Fan, Chunhai
Fan, Ruiqing
Fan, Wei
Fan, Xue-sen
Fan, Zhijin
Fanali, C.
Fañanas, Francisco J.
Fang, Evandro Fei
Fang, Jia-You
Fang, Jim
Fang, Jinggui
Fang, Ye
Fang, Yulin
Fanning, Kent J.
Fant, Kristina
Fantus, I. George
Fanucci, Gail E.
Farag, Mohamed A.
Farias, Jorge G.
Farid, Chemat
Farinati, Fabio
Farrell, Robert
Farris, Stefano
Farsky, Sandra H.p.
Farver, Ole
Fasano, Elena
Faucheux, Nathalie
Faucitano, Antonio
Faustino, Amparo
Favero, Gabriele
Favi, Gianfranco
Fazio, Alessia
Feás, Xesús
Federico, Alessandro
Fedin, Matvey
Fedorova, Olga S.
Felix Saavedra, Maria José
Feng, Shilan
Feng, Yangbo
Feng, Yibin
Fenical, William
Fernández-López, Jose
Fernandes-Pedrosa, Matheus de Freitas
Fernandez, Pablo Lodeiro
Fernandez-Lafuente, Roberto
Fernández-Pérez, Leandro
Fernández-Ponce, M. Teresa
Fernando, Pasan
Ferrand, Yann
Ferrari, Erika
Ferrari, Stefania
Ferreira, Isabel
Ferreira, Paula
Ferreira, Vania Maria Moraes
Fett-Neto, Arthur G.
Fiammengo, Roberto
Fiaschi, Tania
Fidele, NTIE KANG
Figadere, Bruno
Filarowski, Aleksander
Filipovic, Milos R.
Filippov, Sergey K.
Fillion, Eric
Finkielsztein, Liliana M.
Finzel, Barry
Fiorentino, Antonio
Fishman, Ayelet
Fitches, Elaine C
Fleisher-Berkovich, Sigal
Flood, Amar
Florea, Bogdan I.
Flores, Pilar
Fock-Bastide, Isabelle
Follmer, Cristian
Fonari, Marina S.
Font, Josep
Fontana, Andreia Cristina Karklin
Fonteh, Alfred N.
Forlani, Giuseppe
Fornasiero, Paolo
Forni, Alessandra
Forsthuber, Thomas G.
Fossen, Torgils
Foster, Brian C.
Foubelo, Francisco
Fouilland, Eric
Fox, Mark
Fraile, Lorenzo
Frairi, Roberto
Francioso, Antonio
Francis, Heather
Franck, Julien
Frank, Éva
Frappier, Lori
Frau, Roberto
Freije, José M.P.
Freitas, Adilson A.
Freitas, Cleverson D. T.
Freitas, Sandra Borges
Fremd, Carlo
Fribley, Andrew M.
Friesen, Duane
Frija, Luís M. T.
Fritz, Torsten
Froldi, Guglielmina
Frolova, Lilya
Frontera, Patrizia
Froudakis, George E.
Frutos, Maria Jose
Fu, Hua
Fu, Ming-Lai
Fu, Qiang
Fu, Tzu-Fun
Fu, Wei
Fu, Yong-Bi
Fujihara, Shinobu
Fujii, Takeshi
Fujimori, Ko
Fujita, Masayuki
Fujiwara, Kenshu
Fukaya, Takayuki
Fukuyama, Takahide
Fukuyama, Tomoki
Fulop, Ferenc
Fuly, André Lopes
Fung, Eileen
Fuqua, Josha
Furuta, Takumi
Fusi, F.
G. Reinecke, Manfred
Gaboriaud, DrNicola
Gagnon, Alexandre
Gahan, Lawrence R.
Gailing, Oliver
Gait, Michael J.
Galdiero, Massimiliano
Galeazzi, Roberta
Galeone, Aldo
Galiano, Silvia
Gali-Muhtasib, Hala U.
Gallo, Alessandra
Gallou, Fabrice
Gallyas, Ferenc
Galvan, Marta
Galvez, Eva M.
Gambaryan, Stepan
Gambin, Yann
Gameiro, Paula
Gámez-Meza, N.
Gan, Kim-Hong
Ganesh, V. Anand
Gangfeng, Ouyang
gannett, Peter
Gao, Feng
Gao, Hao
Gao, Jing
Gao, Jinming
Gao, Kun
Gao, Ruili
Gao, Wei
Gao, Yi Qin
Garau, Alessandra
Garbis, Spiros
García Fernández, José Manuel
García, Abraham
Garcia, Antonio
García, Hermenegildo
García-Álvarez, Joaquín
García-Beltrán, Olimpo
García-Caballero, Melissa
García-Gonzáles, Rolando
Garcia-Granda, Santiago
García-Sánchez, Miguel A.
García-Villanova, Belén
García-Vivó, Daniel
Gariboldi, Manuela
Garman, Scott C
Garozzo, Domenico
Garro, Marisa S.
Garrote, Gil
Gaspar, Diaz
Gasser, Christoph
Gattuso, Giuseppe
Gaunitz, Frank
Gavicho Uarrota, Virgilio
Gazouli, M.
Gbaguidi, A. Fernand
Ge, Yubin
Geary, Sean
Gebicki, Janusz
Gehrke, Sebastian S.
Geraghty, Dominic P.
Geronikaki, Athina
Gerothanassis, Ioannis P.
Gerschenson, Lía Noemí
Gervay-Hague, Jacquelyn
Gerwick, Cliff
Ghafoor, Abdul
Ghawi, Sameer Khalil
Ghosh-Choudhury, Nandini
Giampieri, Francesca
Giancane, G.
Giannini, Giuseppe
Gibbons, Simon
Gibson, Matthew
Giese, Bernd
Gijzen, Mark
Gikas, E.
Gillies, Elizabeth
Gillinham, Dennis
Gilmer, John F.
Gilmore, TD
Gil-Ramírez, Alicia
Gimenez, Sixto
Gimeno, Miquel
Giner-Casares, Juan J.
Gioiello, Antimo
Giorgi, M.
Giorio, Giovanni
Giovannini, Pier Paolo
Giraldo, Liliana
Girard-Thernier, Corine
Giraudeau, Patrick
Girbés, Tomás
Girousi, Stella
Giudice, Aldo
Gizdavic-Nikolaidis, Marija R.
Glaser, Keith B.
Glatt, H.
Glue, Paul
Gnanasekar, Munirathinam
Gniewosz, Małgorzata
Gobis, K.
Goda, Ahmed E.
Godoy, Sergio Medina
Godt, Adelheid
Gofferjé, G.
Göksu, Süleyman
Goławska, Sylwia
Goldberg, Alfred
Goldstein, Irwin
Goltsov, Alexey
Gomes, Fernanda
Gomes, Sónia
Gómez Di Marco, Perla
Gómez, Irene Crespo
Gómez, Nazely Diban
Gómez, Rafael
Gómez-Caravaca, Ana María
Gómez-Gómez, Lourdes
Gómez-Hens, A
Gómez-Ruiz, Santiago
Gonçalves, G.m.s.
Gonçalves, L.R.B.
Gonçalves, M. Sameiro T.
Gong, Ping
Gong, Xingchu
Gong, Young-dae
González, Concepción C.
Gonzalez, Daniel R
Gonzalez, M. S.
Gonzalez, Mercedes
González-Baró, Ana C.
González-Bello, Concepción
González-Maeso, Javier
González-Mas, M. Carmen
González-Muñiz, Rosario
González-Trujano, Ma. Eva
Gordon, John C.
Gordon, Keith C.
Gordon, Michael
Gorman, Gregory S
Gorrell, M. D.
Gorski, Waldemar
Gorzalczany, S.
Gosmann, Grace
Gostner, Johanna M.
Gotor-Fernández, Vicente
Gou, Shao-Hua
Goulas, Vlasios
Goumont, Régis
Govindarajulu, Rajanikanth
Gowen, Aoife
Goya, Luis
Gozzo, Franco
Grace, Mary
Granica, Sebastian
Grassi, Alfonso
Grassi, Giovanni
Graton, Jérôme
Grattoni, Alessandro
Gravel, Valerie
Gray, Christopher A.
Gray, Derek G.
Grayer, Renée J.
Grée, René
Greenberg, Robert M.
Greenberger, Joel S.
Greenspan, Phillip
Greenwood, Michael T.
Grgurić-Šipka, Sanja
Grienke, Ulrike
Grigalevicius, Saulius
Grigg, Ronald
Grigoriev, I. P.
Grill, Leonhard
Grimi, Nabil
Grimley, Philip M.
Grindley, T. Bruce
Grose, Julianne H
Gruber, Christian
Gruner, Bohumir
Gruss, Alexandra
Grüttner, Cordula
Grzebisz, Witold
Gu, Jianguo
Gu, Man Bock
Gu, Weikuan
Guddat, Luke W.
Guerin, Marcelo E.
Guerra, Francisco M.
Guetet, Arbi
Gueyrard, David
Guga, Piotr
Guidolin, Diego
Guilhon, Giselle Maria S. P.
Guimaraes Leitão, Gilda
Guisan, Jose Manuel
Gulaboski, Rubin
Gunaje, Jayarama
Guo, Fenghai
Guo, Grace
Guo, Lian-Wang
Guo, Xinwen
Guo, Yuanqiang
Guo, Zhanhu
Guo, Zheng
Gupta, Vinay
Gurzov, Esteban N.
Gust, Ronald
Gutiérrez-Uribe, Janet A.
Ha, Hyun-Joon
Ha, Tae-Yeal
Haba, Hamada
Hackenberg, Michael
Hadjikakou, Sotiris K.
Hadjipavlou-Litina, Dimitra
Haenisch, Sierk
Haertlé, Thomas
Hager, Martin D.
Hagström, ÅK
Hahm, Eun-Ryeong
Hahm, Ki-Baik
Hai, Faisal Ibney
Haider, Norbert
Hak, Sjoerd
Hakamata, Hideki
Halabalaki, Maria
Haleagrahara, Nagaraja
Hall, Edward D.
Haltrich, Dietmar
Hamazaki, Kei
Hamberger, Björn
Hamblin, Michael R.
Hamdane, Djemel
Hamdi, Naceur
Hameed, Shahid
Hammadeh, M.
Han, Bing
Han, Hogyu
Han, Jin
Han, Quan-Bin
Han, Sang-Bae
Han, Shiqing
Han, Yue-Peng
Hanafy, Khalid
Handy, Scott T.
Hanks, Timothy
Hansen, Michael Ryan
Hansen, P.E.
Harbourne, Niamh
Harding, James
Harju, Anni
Harlev, Eli
Haroutounian, Serkos A.
Harrison, William
Hart, Joanne
Hartley, Thomas
Hartmann, Martin
Harvey, Alan
Hasegawa, Makoto
Hasegawa, Urara
Hashimoto, Masaru
Hassan Gilani, S.I., Anwarul
Hassan, Fathi
Hasselmann, Dieter
Hatano, Tsutomu
Haufe, Günter
Hay, Stephen O.
Hayashi, Ken-ichiro
Hayashi, Minoru
Hayashi, Yoshihiko
Hayes, John D.
Hayes, Maria
Haynes, Laura M.
He, Bin
He, Bingfang
He, Hongwu
He, Jian-Uog
He, Jun
He, Liangnian
Heaney, Frances
Hearn, Michael J.
Hebert, Daniel
Hedstrom, Lizbeth
Heemstra, Jennifer M.
Heger, Michal
Heilemann, Mike
Heinrich, Timo
Heiss, Elke
Helaja, Juho
Helfenstein, Fabrice
Hell, Rüdiger
Hellens, Roger
Hellström, Jarkko
Hendrich, Suzanne
Henle, Thomas
Hennebelle, Thierry
Hensel, A.
Herbert, Cristan
Hermosín-Gutiérrez, Isidro
Hernández Ledesma, Blanca
Hernández, Francisca
Hernandez-Hierro, José Miguel
Herrador, M. Mar
Herraiz, T.
Herranz, Rosario
Herrero-Martínez, José Manuel
Herrmann, Martin
Hesse, Stéphanie
Hiblot, Julien
Hidari, Kazuya I.p.j.
Hideg, Éva
Hideg, Kálmán
Higashi, Shouichi
Hikawa, Hidemasa
Hillard, Elizabeth A.
Himi, Eiko
Hinkula, Jorma
Hipkiss, Alan R.
Hirano, Koji
Hirasawa, Noriyasu
Hirata, Hirokuni
Hiroaki, Hidekazu
Ho, Feng-Ming
Ho, Junming
Ho, Tin-Yun
Ho, Yunn-fang
Höbartner, Claudia
Hocher, Valérie
Hodges, Robert
Hoelder, Swen
Hofer, Tim
Hofmann, Alan F.
Hofmann, Bettina
Hofmann, Ute
Hogg, Philip
Hohlfeld, Thomas
Hohng, Sungchul
Hojo, Keiko
Holder, Alvin A.
Holdt, Hans-Jürgen
Holzgrabe, Ulrike
Hong, Xuechuan
Honma, Tetsuo
Honoré Hansen, Steen
Hood, Matthew A.
Hoogenboom, Bart
Hook, Ingrid L.I.
Horbowicz, M.
Horbowicz, Marcin
Hori, Kanji
Hormi, Osmo
Horobin, Richard W.
Horstkorte, Rüdiger
Hort, Mariana Appel
Hosmane, Ramachandra S.
Hosni, Karim
Hosono, Masahiro
Hosoya, Takashi
Hossain, Mohammad Billal
Hostetler, Heather A.
Ho-Tin-Noé, Benoît
Hou, Chien-Wei
Hou, Tingju
Hou, Tingjun
Hou, Weihong
Houen, Gunnar
Houng, Jer-yiing
Hour, Mann-Jen
Hoving, J. Claire
Howitt, Crispin A.
Hrelia, Silvana
Hsiao, George
Hsiao, Pei-Wen
Hsieh, Pei-wen
Hsieh, Yi-Hsien
Hsieh, Yves
Hsing, Chung-Hsi
Hsu, Hsu
Hsu, Kuo-Chiang
Hu, Deyu
Hu, Guoku
Hu, Jiang
Hu, MingKuan
Hu, Ping
Hua, Erbing
Hua, Susan
Huang, Beibei
Huang, Chi-Chang
Huang, Chih-Ching
Huang, Hui-Yu
Huang, Jianliang
Huang, Jui-Hsien
Huang, Jun
Huang, Keng-Shiang
Huang, Lin-Fang
Huang, Linjuan
Huang, Tien L.
Huang, Wei-Jan
Huang, Wen-Hsin
Huang, Wenlong
Huang, Xiao-Jun
Huang, Xinchuan
Hubbard, Rod
Huck, Christian
Huczyński, Adam
Hudhomme, Piétrick
Hudson, Robert
Huefner, Antje
Huffaker, Alisa
Hugueney, Philippe
Huh, Do Sung
Huh, Man Kyu
Huh, Tae-Lin
Hulme, Christopher
Hung, Li-man
Hunsche, Mauricio
Huq, Fazlul
Hurvois, Jean-pierre
Hussein, Ahmed A.
Hussein, Waleed
Huynh, Thuan Minh
Hwang, Bang Yeon
Hwang, Dae Youn
Hwang, Geum-Sook
Hwang, Ki-chul
Hwang, Tsong-Long
Iacobellis, Nicola S.
Iannello, Carmelina
Ichinose, Koji
Ida, Elza Iouko
Igarashi, Kiharu
Igarashi, Miki
Igau, Alain
Iglesias, Marta
Iglesias, Rosario
Iida, Takashi
Iino, Ryota
Ilari, Andrea
Ilic, Nebojsa
Im, Wan-Taek
Imanishi, Akihito
Imberty, Anne
Inagaki, Masaki
Ingram, Susan L.
Inkster, James
Innocentini Mei, Lucia Helena
Inokuchi, Tsutomu
Inoue, Hiroyasu
Insuasty, Braulio
Ionel, Mangalagiu
Iorizzi, Maria
Iorizzo, Massimo
Iossifidou, Eleni
Iriti, Marcelo
Isaad, Jalal
Ishibashi, Masami
Ishihara, Kazuyuki
Ishikura, Minoru
Isidoro, Ciro
Isman, Murray B.
Isobe, Sachiko
Isshiki, Yoshiaki
Ito, Michiho
Ito, Yoichiro
Itoh, Nobuya
Itoh, Tomohiro
Ivanov, Ivo C.
Ivanova, Bojidarka
Ivanova, Elena P.
Iwai, Naoharu
Iwan, Agnieszka
Iwao, Masatomo
Iwasaki, Tomohiro
Iwashina, Tsukasa
Iwata, Tadahisa
J Martín-Rodríguez, Alberto
Jáč, Pavel
Jackson, William F.
Jacob, Claus
Jacobsen, Elisabeth Egholm
Jacquot, Yves
Jaffrezic-Renault, Nicole
Jagerovic, Nadine
Jakob, Ursula
Jakubowski, Joseph A
Jameson, David
Jampilek, Josef
Janetka, James W.
Janfelt, Christian
Jang, C. S.
Jang, Dae Sik
Jang, Hae-Dong
Jang, Hyeung-Jin
Jang, Jinho
Jantas, D.
Jarret, Robert L.
Jasinski, Jerry P.
Je, Jae-young
Jeandet, Philippe
Jefferies, L.K.
Jeffrey, Christopher S.
Jelen, Henryk
Jenks, William
Jensen, Søren Rosendal
Jensen, Svend
Jeon, Byeong Hwa
Jeon, Ju-hong
Jeong, Byeongmoon
Jeong, Ill Yun
Jeong, Lak Shin
Jerković, Igor
Jerz, Gerold
Jeschke, Gunnar
Jesionowski, Teofil
Jeyaseelan, Kandiah
Ji, Bin
Ji, Hai-Feng
Ji, Nai-Yun
Ji, Tengfei
Jia, Li
Jia, Zhenquan
Jiang, De An
Jiang, Guang
Jiang, Guoqiang
Jiang, Lin
Jiang, Ming
Jiang, Yueming
Jiang, Yuyang
Jiao, Wanting
Jimbo, Mitsuru
Jiménez, Ana
Jiménez-Barbero, Jesús
Jimenez-Flores, Rafael
Jimenez-Lopez, Jose
Jin, Tienan
Jindrich, Jindrich
Jing, Dengwei
Jing, Jing
Jiráček, Jiří
John A., Beutler
Johnson, Jeremy J.
Johnston, Carl Gordon
Johnston, Carol
Jolivalt, Corinne G.
Jonnala, Surendranadha Reddy
Joo, Choun-Ki
Jordan, Joaquin
Jordão, António M.
Jørgensen, Kåre
Joshi, Tanmaya
Jou, Jwo-Huei
Jovanovic, Jelena D.
Joven, Jorge
Joy, Margarita
Jóźwiak, Krzysztof
Juers, Douglas H.
Juhasz, Bela
Juliá, Luis
Jung, Hyun Ah
Jung, Klaus
Jung, Sang Hun
Junker, Jan Philipp
Jurado, Amália S.
Jurikova, Tunde
Juskowiak, Bernard
Juszczak, Lesław
Juvik, John
K. Deyholos, Michael
Kaczor, Agnieszka
Kaczorek, Ewa
Kad, Neil M.
Kadokawa, Jun-ichi
Kaeko, MUROTA
Kafarski, Pawel
Kai, Guoyin
Kakiyama, Genta
Kakkar, Ashok K.
Kalinin, Stanislav
Kalinin, Vladimir
Kalinowska-Lis, Urszula
Kaliora, Andriana C.
Kallunki, Tuula
Kalska-Szostko, B.
Kaltner, Herbert
Kamei, Junzo
Kamel, Alaa
Kamenecka, Theodore M.
Kaminska, Bozena
Kan, Chi
Kanai, Masashi
Kanan, Yogita
Kanaoka, Masahiro
Kaneco, Satoshi
Kaneda, Kiyotomi
Kaneko, Kimiyuki
Kang, Chulhun
Kang, Hee Eun
Kang, Jong
Kang-Mieler, Jennifer J.
Kanie, Osamu
Kao, Ming-Ching
Kao, Hsien-ming
Kappe, C. Oliver
Karakhanov, Edward
Karimi, Mahsan
Karioti, Anastasia
Karoyan, Philippe
Karpoormath, Rajshekhar
Kaschula, Catherine H.
Kashfi, Khosrow
Kashiwada, Yoshiki
Katagiri, Hiroshi
Katarg, Argyropoulou
Katayama, Hidekazu
Kato, Katsuya
Kato, Koichi
Kato, Tatsuya
Katz, Jonathan I.
Kaufmann, Dieter
Kauhaluoma, Jari
Kawabata, Kyuichi
Kawahara, Teppei
Kazem, Ahmed
Ke, Xuebin
Ke, Yanxiong
Keating, David H.
Keglevich, György
Keijitsu, Keijitsu
Keil, Frerich
Keller, Paul A.
Kellow, NJ
Kelly, Charles
Kennedy, Eileen J.
Kenyon, William J.
Kerwin, Sean M.
Keum, Gyochang
Kevil, Christopher
Keyzers, Rob
Khan, M. Akram
Khan, Nafees
Khiari, Zied
Kiba, Akinori
Kiec-Kononowicz, Katarzyna
Kieffer, Bruno
Kietzmann, Thomas
Kikuzaki, Hiroe
Kilian, Stefanus
Kim, Guncheol
Kim, Hae-Jo
Kim, Harold
Kim, Hee-Sun
Kim, Hyeung-rak
Kim, Jeong-Han
Kim, Jin-hyun
Kim, Jin-Kyung
Kim, Jung-ae
Kim, Keun-Sung
Kim, Ki Hyun
Kim, Kil-Soo
Kim, No Soo
Kim, Sang Kyum
Kim, Sehoon
Kim, Seung Hyun
Kim, Sun Chang
Kim, Sung Hoon
Kim, Sunghoon
Kim, Yangmee
Kim, Yeong-Shik
Kim, Yeon-Ju
Kim, Yong Hwan
Kim, Young Kyoo
Kim, Yun-bae
Kim, Yuri
Kimura, Hideo
Kimura, Hideto
Kimura, Masanari
King, Charles C.
King, Frank D.
King, Jerry
King, Petra
Kinugawa, S.
Kiparissides, C.
Kireev, Dmitri
Kirsch, Gilbert
Kirsch, Thorsten
Kirst, Herbert A
Kisch, Horst
Kitade, Yukio
Kitagawa, Seiichi
Kjems, Jørgen
Klapötke, Thomas
Klauber-DeMore, Nancy
Kleij, Arjan W.
Klemm, Dieter
Kletsas, Dimitris
Klettner, Alexa
Klinkhamer, Peter G.L.
Kljun, Jakob
Klode, Joachim
Klotz, Lars-Oliver
Klymchenko, Andrey
Knauer, Shirley K.
Knežević, Nikola Ž.
Knölker, Hans-Joachim
Knupp, Gerd
Ko, Kam Ming
Kobayashi, Isao
Kobayashi, Kazuhiro
Kobayashi, Kazuo
Kobayashi, Shizu
Kobayashi, Yuichi
Kobori, Akio
Kobori, Masuko
Kockx, Maaike
Kojima, Akiko
Kojo, Shosuke
Kokotkiewicz, A.
Koksharova, T. V.
Kolehmainen, Erkki
Kolodziejczyk, Joanna
Komaitis, Michael
Komeyama, Kimihiro
Komiyama, Makoto
Kong, In-soo
Kong, Ling-Yi
Kongsted, Jacob
Konig, Helmut
Konrath, Eduardo Luis
Konstantinidis, Stephanos
Kontominas, Michael
Koodali, Ranjit
Koóš, Miroslav
Koppel, Kadri
Korkaya, Hasan
Kornienko, Alexander
Kortner, Trond M.
Kosová, Klára
Kostecka, Małgorzata
Kostogrys, Renata
Koszytkowska-Stawińska, Mariola
Koufaki, Maria
Kourimska, Lenka
Kouyama, Tsutomu
Kovacs, Lajos
Kowalska, Teresa
Koya, Daisuke
Koźmiński, Wiktor
Kragl, Udo
Krajcsi, Peter
Krajewska, Barbara
Krajka-Kużnik, Violetta
Krása, Josef
Krasnoholovets, Volodymyr
Kraus, Tobias
Krauss, Isaac J.
Krawetz, Roman
Kreis, Wolfgang
Křen, Vladimír
Kretzschmar, Georg
Krishnan, Kannan
Krušić, Melina Kalagasidis
Krysinski, Pawel
Kuan, Yu-Hsiang
Kuang, Hai-Xue
Kubáň, Vlastimil
Kubota, Toshiaki
Kubota, Yoshihiro
Kuca, Kamil
Kuda, Takashi
Kuder, Tomasz
Kudo, Yasusei
Kukovecz, Ákos
Kulma, Anna
Kumamoto, Eiichi
Kumar, Anil
Kumar, Rakesh
Kumar, Satish
Kumirska, Jolanta
Kuno, Toshiya
Kunz, Horst
Kuo, Jen-min
Kuo, Ping-chung
Kuramochi, Kouji
Kurlyandskaya, Galina
Kurz, Thomas
Kusakabe, Ken-ichi
Kveberg, Lise
Kwasniewski, Misha
Kwiecien, Slawomir
Kwon, Oran
Kwon, Sung Won
Kwon, Youngjoo
La Clair, James
La Rocca, Renato V.
La, Hyun-Joon
Laali, Kenneth K.
Lacaille-Dubois, Marie-Aleth
Lacrampe, Marie France
Ladero, M.
Lagarde, Florence
Lai, Hsi-Mei
Lai, Maoyi
LaJeunesse, Dennis R.
Lakhtin, V.
Lakshmanan, Venkatachalam
Lallès, Jean-Paul
Lam, Henry
Lamberth, Clemens
Lameira, Jerônimo
Lan, Wen-Jian
Lanari, Daniela
Lantto, Perttu
Lapres, John J.
Laquintana, Valentino
Lara-Sanchez, Agustin
Laszlo, Joseph A.
Laufer, Stefan
Lautenschläger, Christian
Lavasanifar, Afsaneh
Lawson, Charlotte
Lay, Horng-Liang
Lazos, Evangelos S.
Lazzarato, Loretta
Leccese, Annamaria
LECLÈRE, Philippe
Lecomte, Philippe
Lee Suan, Chua
Lee, Adam
Lee, Bao-Shiang
Lee, Bruce P.
Lee, Che-Hsin
Lee, Cheng-I
Lee, Choon Young
Lee, Choong Hwan
Lee, Dong-ung
Lee, Dongwon
Lee, Duu-Jong
Lee, Hian Kee
Lee, Hon Man
Lee, Hsin-Chen
Lee, Hsinyu
Lee, Hye Suk
Lee, Jae Kwon
Lee, Jeong-chae
Lee, Jeongmi
Lee, Jin Hyung
Lee, Jin-ching
Lee, Jung Heon
Lee, Jung-Kul
Lee, Jungmin
Lee, Lin-wen
Lee, Min Won
Lee, Moses
Lee, S. Yong
Lee, Sang Gil
Lee, Sang Kook
Lee, Sang-Soo
Lee, Seokjoon
Lee, Seong-Ryong
Lee, Seung Ju
Lee, Shyi-Long
Lee, Sunwoo
Lee, Sun-Young
Lee, Tu
Lee, Tzong-Huei
Lee, Tzong-shyuan
Lee, Won Sup
Lee, Woo Song
Lee, Yong Rok
Lee, Young Sup
Lee, Zang Hee
Leem, Kang-Hyun
Leenders, William P.J.
Legendre, Laurent
Lehle, Karla
Leidinger, Petra
Leitner, Erich
Leivonen, S.K.
Lemkul, Justin A.
Len, Christophe Len
Lenth, Russell V.
Léonel, Eric
Leonetti, Francesco
León-Rivera, Ismael
Lepenies, Bernd
Lépine, Francis
Lepore, Maria
Les, Donald H.
Lesinski, Gregory B.
Lesyk, Roman
Leu, Yann-Lii
Leu, Yu-Ling
Leung, A.W.N.
Leung, Chung-Hang
Leung, Elaine Lai-Han
Leung, Man-kit
Leung, Ping-Chung
Lew, D. Betty
Lezcano, J.M.
Lezot, Frédéric
Li, Donghao
Li, Duo
Li, Fengting
Li, George
Li, Guijie
Li, Hua-Bin
Li, Huige
Li, Jian
Li, Jianming
Li, Jianshu
Li, Jinjun
Li, Juan
Li, Ke
Li, Kefeng
Li, Li
Li, Nianguang
Li, P.M.
Li, Ping-Ya
Li, Qi
Li, Shao-Shun
Li, Shiyou
Li, Shu-Ming
Li, Wang
Li, Wei
Li, Xia
Li, Xiansen
Li, Xiaobo
Li, Xingshu
Li, Xingtai
Li, Xuechen
Li, Yu-Teh
Li, Zhiyong
Li, Zibo
Lian, Jane B.
Lian, Xiao-Yuan
Liang, Chengyu
Liang, Chia-Hua
Liang, Hong
Liang, Pi-Hui
Liao, Bo
Liao, Jyh-fei
Liaw, Chih-Chuang
Liby, Karen
Lichtenstein, Rachel G.
Liebau, Eva
Liebscher, Juergen
Lila, Mary Ann
Lim, Beong Ou
Lim, Chee Wei
Lim, Dongwook
Lim, Soon Sung
Lim, Y.M.
Lima-Brito, José Eduardo
Limbach, Hans-Heinrich
Limbach, Michael
Lin, Carol
Lin, Chih-Cheng
Lin, Chih-Ching
Lin, Chiou-feng
Lin, Chun-Cheng
Lin, Chunmian
Lin, Hai-shu
Lin, Hsiang-Ru
Lin, Hui-Hsuan
Lin, Ivan J.B.
Lin, Jing-jer
Lin, Jin-yuarn
Lin, Jun
Lin, Quankui
Lin, Shyh-Hsiang
Lin, Wenhan
Lin, Wey-jinq
Lin, Yun-lian
Lin, Zhenjian
Lin, Zhihua
Lin, Zhixiong
Lincoln, Per
Lindemann, Peter
Lindequist, Ulrike
Lindhorst, Thisbe
Lindsey, Keith
Link, Andreas
Lioi, Lucia
Lionetto, M. G.
Liston, Adrian
Litinas, Konstantinos E.
Little, R. Daniel
Liu, Chang-Xiao
Liu, Chong-Huai
Liu, Dehua
Liu, Delong
Liu, Dongmin
Liu, Fang
Liu, Feng
Liu, Gang
Liu, Haining
Liu, Hai-Yang
Liu, Hsiao-Sheng
Liu, Jiang-Yun
Liu, Jiaren
Liu, Jie
Liu, Jin
Liu, Jingbo
Liu, Jun Xi
Liu, Jun-Jen
Liu, Kexin
Liu, Lei
Liu, Liang
Liu, Liwang
Liu, Minghua
Liu, Qiancai
Liu, Qing-hui
Liu, Qingsong
Liu, Shi J.
Liu, Shing-Hwa
Liu, Shi-Xia
Liu, Shulin
Liu, Wei
Liu, Xinghai
Liu, Xinyong
Liu, Yang
Liu, Ying
Liu, Yonghong
Liu, Yongjun
Liu, Yuanhong
Liu, Yuelian
Liu, Zai-qun
Liu, Zhi Long
Liu, Zhongqiu
Livrea, Maria Antonia
Lizard, Gérard
Lo Faro, Maria Letizia
Lo, Clive
Lo, Jeng-Fan
Lo, Lee-Chiang Lo
Locatelli, Marcello
Locatelli, Monica
Loizzo, Monica Rosa
Lomberget, Thierry
Long, Chao-An
Long, Chunlin
Longo, Pasquale
Longo, Vincenzo
Longobardi, Francesco
López, Concepción
López-Abán, Julio
López-Carballo, Gracia
Lopez-Gallego, Fernando
López-Rodríguez, María L.
Loppi, S.
Lorberboum-Galski, Haya
Lorenzo, E.
Lorkowski, Stefan
Loska, Rafał
Lou, Bih-Show
Lou, Hong Xiang
Lou, Y.J.
Loukovaara, Sirpa
Lovero, Grazia
Lowary, Todd L.
Lowry, Christopher A.
Lozano, Pedro
Lu, Jun
Lu, Kui
Lu, Ling
Lu, Mei-Chin
Lu, Rong
Lu, Wei
Lu, Yinrong
Lu, Zhaoxin
Lucenti, Elena
Ludwiczuk, Agnieszka
Luesch, Hendrik
Lugemwa, Fulgentius Nelson
Luisi, Renzo
Luna, Jonny E. Duque
Luneau, Dominique
Luo, Cheng
Luo, Huan-Min
Luo, Shenglian
Luo, Xiaoxing
Luo, Yinggang
Luo, Zisheng
Lupiáñez, José A.
Lupidi, Giulio
Lurie, Susan
Lustri, Wilton Rogério
Luzhkov, Victor B.
Lygate, Craig A.
Lykakis, Ioannis
Ma, Bai-ping
Ma, Chaomei
Ma, Chen
Ma, Edmond Dik-Lung
Ma, F.
Ma, Jin Yeul
Ma, Wanhong
Ma, Yu-qiang
Ma, Zhong-Jun
Macaluso, Filippo
MacCallum, Donna
Machon, Denis
MacKenzie, Donald
Mackenzie, Graham
Maćkowski, Sebastian
Macyk, Wojciech
Maddirela, Dilip R.
Madureira, João
Maeda, Isamu
Maeda, Toshinari
Mafu, Sibongile
Magda, Jules J.
Maggi, Filippo
Magid, Abdulmagid Alabdul
Magliozzo, Richard
Magnani, Mauro
Mahdi, Jassem
Mahelka, Václav
Maher, Pamela
Mahiou-Leddet, Valérie
Mahmood, Khalid
Mahrwald, Rainer
Maia, José Guilherme S.
Maioli, Emanuela
Majewsky, Marius
Major, Marian E.
Majumdar, Susruta
Makala, Levi HC
Makino, Toshiaki
Malan, Sarel F.
Malde, Alpesh
Maldonado-Medina, Rosa Amelia
Malekinejad, H.
Malfa, Stefano La
Malki, Mustapha
Mallapragada, Surya K.
Malmsten, Martin
Maloney, Mark
Mambu, Angele
Mambu, Angèle Lengo
Man, Kwan
Mancheño, José
Mancheño, Olga García
Mancuso, Cesare
Mancuso, Raffaella
Manenti, Stephane
Manganaris, George A
Mangu, Naveen
Mann, Enrique
Mannini, Matteo
Mansoori, G. Ali
Mantell, C.
Manthey, John A.
Mantovani, Alberto
Mantzavinos, Dionissios
Manzhos, Sergei
Mao, Hanbin
Mao, Xiangzhao
Maoz, Ben
Marangoni, Franca
Marasco, Daniela
Marco-Contelles, José
Marie, Emmanuelle
Marik, Jan
Marimpietri, Danilo
Marin, Jose J. G.
Marin, Luminita
Marín, Sonia
Marinesco, Stéphane
Marino, Angela
Marison, Ian
Marken, Frank
Marković, Zoran
Marmion, Celine J.
Marques, Fernanda
Marques, José C.
Marreilha Dos Santos, Ana Paula
Marsh, Deborah
Marsich, Eleonora
Martel, Fátima
Martens, Craig C.
Marti, Guillaume
Martí, Sérgio
Martín, A.
Martin, Daniel
Martin, Julia
Martín, Pablo Antonio Lara
Martinelli, Adriano
Martínez, Ana M.
Martinez, Gary V.
Martínez-Díaz, Rafael A.
Martínez-Palou, Rafael
Martinoli, Maria-Grazia
Martin-Ortigosa, Susana
Martins, Filomena
Martins, Luísa M. D. R. S.
Martins, M. R.
Martins, Rosário
Marx, Andreas
Marzouk, Amani M.
Mashtoub, Suzanne
Maskrey, Benjamin H.
Mason, Ronald
Mastino, Antonio
Masu, Hyuma
Masuoka, Noriyoshi
Materska, Małgorzata
Mateus, Nuno
Mathé, Christophe
Mathesius, Ulrike
Matkowski, Adam
Matos, Maria Joao
Matsubara, Kouki
Matsuguchi, Tetsuya
Matsui, Takuya
Matsui, Toshiro
Matsumoto, Kozo
Matsumoto, Yoshimi
Matsunami, Katsuyoshi
Matsuo, Yukiko
Matthaei, Hanno
Matthews, Paul D.
Matyus, Peter
Mavromoustakos, Thomas
Mayer, Christian
Mazorra-Manzano, Miguel A.
Mazutti, Marcio A.
Mazzei, Franco
Mazzoni, I.
McAlpine, Shelli
Mcanulty, Steve
McCallum, Jason L.
McCann, Mark J.
Mccarthy, Peter
McCarty, Owen
Mcconnell, Michelle
Mcdowell, Arlene
McGill, Mitch
Mcginley, John
Mcinnes, Campbell
McMenamin, Paul Gerard
McMullin, David R.
McPhillie, Martin
Medina, Miguel
Medina, Miguel Ángel
Medina, Roxana
Mekhloufi, Ghozlene
Melikov, Kamran
Melino, Sonia
Menaa, Farid
Mena-Rejon, Gonzalo J.
Mendes, Adriano Aguiar
Mendez, Luciana
Méndez-Ardoy, Alejandro
Menendez, Jose Carlos
Menéndez, Pilar
Menezes, José
Meng, Fanhao
Meng, Fenghua
Meng, Qingxiong
Menkissoglu-Spiroudi, Urania
Menna, Marialuisa
Mercader, Andrew G.
Merín, María Gabriela
Merino-Gergichevich, Cristian
Merlini, Lucio
Mesías, Marta
Mesmaeker, Andrée Kirsch-De
Messeguer, Angel
Messori, Luigi
Meyer-Hamme, Gesa
Mezzetti, Bruno
Mi, Man-tian
Miao, Jun-ying
Miceli, Antonio
Michaelakis, Antonios
Micheli, L.
Michellys, Pierre-Yves
Micura, Ronald
Miguel, M. G.
Mikata, Yuji
Mikkonen, Kirsi S.
Miklós, Mézes
Mikstacka, Renata
Mikulski, Damian
Mikusova, Katarina
Milella, Luigi
Milelli, Andrea
Milenkovic, Dragan
Miller, Anthony B.
Miller, Marshall G.
Milojković-Opsenica, Dušanka M.
Milstein, David
Mimica-Dukic, Neda
Mimmo, Tanja
Min, Kyunghoon
Min, Sun-joon
Minbiole, Kevin P. C.
Minehan, Thomas G.
Minoura, Mao
Miranda, Katrina
Miranda, L.
Miret-Artés, Salvador
Mirica, Liviu M.
Misaka, Shingen
Mischne, Mirta P.
Misle, Enrique
Mita, Tsuyoshi
Mitakou, Sofia
Mitronova, Gyuzel
Miyabe, Hideto
Miyagishi, Makoto
Miyata, Yoshihiko
Miyazaki, Kouji
Miyazaki, Masaya
Mizuno, Cassia S
Mladěnka, Přemysl
Mlcek, Jiri
Mlinarić-Majerski, Kata
Młochowski, Jacek
Moberg, Christina
Mocanu, G.
Mohan, Tamilselvan
Mohd Esa, Norhaizan
Möhwald, Helmuth
Moineau-Chane-Ching, Kathleen I.
Mojtahedi, Mohammad M.
Mojžiš, Ján
Mok, Daniel Kam-Wah
Molinari, Alessandra
Molinari, Francesco
Moliner, Manuel
Mollapour, Mehdi
Mollica, Adriano
Molmeret, Maëlle
Molnar, Maja
Molnár, Péter
Monchaud, David
Monforte, Antonio Jose
Monnier, Florian
Monsees, T. K.
Montenegro, Lucia
Montero, Mayte
Montes, Antonio
Montes, Juan
Montes, Vince
Moodley, Brenda
Moo-Huchin, Víctor M.
Moon, Hyung-In
Moon, Jeon-Ok
Moor, Cornelia H. de
Moore, John
Moorthy, N.S. Hari Narayana
Morales, M. P.
Moran, Jeffery H.
Moravcová, Jitka
Morcos, Nabil
Moreno, Diego
Moreno, Silvia
Moretto, Alessandro
Morgan, Kevin
Mori, Naoki
Mori, Yuji
Morikawa, Toshio
Morimura, Shigeru
Moriwaki, Hiroshi
Morreale, Marco
Morris, Gordon A
Morris, Jonathan
Morris, Viola B.
Morschhäuser, Roman
Mosca, Simone
Mossialos, Dimitris
Mostafavi, Mehran
Mott, Ivan
Moujir, L. Moujir
Moutsatsou, P.
Moyano, Albert
Mphahlele, Malose Jack
Muccioli, Giulio G.
Mueller, Jens
Muhle, Claudia
Mukudai, Yoshiki
Mullen, Kathleen M.
Muller, C.j.f.
Muller, Christa
Mullins, Robert F.
Mulvihill, Erin
Muñiz-Salazar, Raquel
Muñoz-Bonilla, Alexandra
Muñoz-Torrero, Diego
Murafuji, Toshihiro
Murakami, Hiroshi
Murakami, Kazuma
Murakami, Manabu
Muramoto, Koji
Murata, Kenji
Murray, Eoin
Murru, Siva
Musa, Musa
Musci, M.
Musco, Giovanna
Musiol, Robert
Muszyńska, Bożena
Muthukrishnan, Subbaratnam
Myung, Chang-seon
Myung, Ja Hye
Myung, Pyung-Keun
Manojlovic, N. T.
Na, Dong Hee
Nadhlala, A. R.
Nag, Arup
Nagai, Kouhei
Nagai, Ryoji
Nagasawa, Kazuo
Nagy, Istvan
Nagy, Zsombor Kristof
Nakagawa, Kyuya
Nakamura, Itaru
Nakamura, Seiichi
Nakamura, Seikou
Nakamura, Yoshimasa
Nakata, Rieko
Nakatani, Hisayuki
Nalewajko-Sieliwoniuk, Edyta
Namba, Kosuke
Nasri, Rim
Natarajan, Viswanathan
Nathan, Ilana
Navarra, Michele
Navarro, Alfons
Navickiene, Sandro
Nayak, Balunkeswar
Nazaruk, Jolanta
Nazinuddin, B.
Nazzaro, Filomena
Ndip, Edmund
Nebbioso, Marcella
Nechab, Malek
Nedachi, Taku
Negron-Silva, Guillermo E.
Nelson, Peter
Nemykin, Victor
Nerín, C.
Nerli, Bibiana B.
Netto, F. M.
Neugart, Susanne
Neumann, Donna M.
Neumann, Terrence
Neuzil, Jiri
Neves, José Manuel
Neves, M. Graça P.M.S
Newman, David
Newman, Stuart
Nguyen, Phuong
Nguyen, Thanh Binh
Nicasio, M. Carmen
Nicholl, Iain
Nickels, Joseph T
Nicolopoulos, Stavros
Niedzwiedzki, Dariusz M.
Nielsen, Poul
Nihei, Ken-ichi
Nikitina, Viktoriya A.
Nikolic, Dejan
Nikonowicz, Edward P.
Nishi, Mayumi
Nishihara, Yasushi
Nishina, Yuta
Nishitani, Yosuke
Nishiwaki, Nagatoshi
Niu, Xiao-Feng
Nobili, Stefania
Noguer, Thierry
Nomikos, Tzortzis
Nomiya, Takuma
Norambuena, Andrés
Norimoto, Hisayoshi
Norte, Manuel
Nosaka, Yoshio
Notni, Johannes
Nouso, Kazuhiro
Novak, Lajos
Novellino, Ettore
Novo, Salvatore
Nowak, Renata
Nowicka, Anna
Nowicka, Anna M.
Nowicki, Janusz
Ntalli, Nikoletta G.
Nurisso, Alessandra
Obata, Toshihiro
Obenland, David
Oberlies, Nicholas H.
Obreza, Aleš
Ochiya, Takahiro
Ochoa, Garcia
Ochoa-Alejo, Neftali
Oda, Tatsuya
Odell, Luke R.
Odero-Marah, Valerie A
O'Doherty, George
Ogawa, Akiya
Ogawa, Shuichi
Oger, Phil M.
Ogoshi, Sensuke
Ogris, Manfred
Ogungbe, Ifedayo Victor
Oh, Hyuncheol
Oh, Sei-Ryang
Oh, Won Keun
Oh, Won-chun
Ohara, Kazuaki
Ohkohchi, Nobuhiro
Ohno, Shinya
Ohta, Masaya
Ohta, Yoshiji
Oikawa, Daichi
Oikawa, Shinichi
Ojika, Makoto
Ok, Salim
Oka, Masahiko
Oka, Natsuhisa
O'Keefe, Barry R.
Okovytyy, Sergiy
Oku, Hirosuke
Okuda, Masahiro
Olafsdottir, Elin Soffia
Olennikov, D. N.
Oliart-Ros, Rosa María
Oliveira, Cristina
Oliveira, Elisabete
Oliveira, P.A.
Oliveira, Rui
Ollis, David
Olmo, Esther Del
Olsen, Christian
O'malley, Bert, W
Omboni, Stefano
Ong, Eng Shi
Ono, Masateru
Oottikkal, Shameema
Ooyama, Yousuke
Oppenheimer, Steven B.
Orčić, Dejan
O'Reilly, Elaine
Ormazábal-Toledo, Rodrigo
Orozco, Fabiola Gutierrez
Orrego, Carlos
Ortega, Angel L.
Ortiz, Maria J.
Osada, Kyoichi
Osheroff, Neil
Østergaard, Jesper
Osterloh, Frank
Oszmiański, Jan
Otsuka, Hideaki
Otsuka, Motoyuki
Ott, Ingo
Ott, Maria
Ottmann, Christian
Ottosson, Henrik
Ouali, Armelle
Ovaska, Timo V.
Oyedeji, Adebola
Pace, Elisabetta
Pace, Vittorio
Padilla, Guillermo
Padilla-Martínez, Itzia I.
Paduano, Luigi
Pae, A.N.
Pae, Hyun-Ock
Pagniez, Fabrice
Pakulski, Zbigniew
Palage, Mariana
Palazon, Javier
Palenzuela López, José Antonio
Palmer, Mike
Palomar, Manuel
Palomo, Claudio
Palou, L.
Palumbo, Gaetano
Pan, Can-ping
Pan, L. K.
Pan, Min-Hsiun
Pan, Qiuhong
Pan, Y. J.
Pan, Yuanjiang
Panacek, A
Panayiotidis, Mihalis I.
Pandanaboina, Sahitya Chetan
Panee, Jun
Paneth, Agata
Panossian, Zehbour
Pant, Deepak
Panza, Luigi
Panzella, Lucia
Papagiannopoulou, Dionysia
Papaioannou, Dionissios
Papaleo, Elena
Papanikolaou, Seraphim
Papenbrock, Jutta
Papetti, Adele
Papy-Garcia, Dulce
Paradiso, Angelo
Paraskevopoulou, Adamantini
Parella, Teodor
Parente, Augusto
Parinandi, Narasimham L.
Park, Il-Kwon
Park, Ji-Yeon
Park, Ki Hun
Park, Kyoung-Chan
Park, Pyo-Jam
Park, Seung-chun
Park, Yong Sun
Parquette, Jon R.
Parra, Roberto
Parveen, Ifat
Paschke, Reinhard
Passarella, Daniele
Pastori, Nadia
Patarrão, Rita
Patel, Mrudula
Pati, Sandra
Paton, Robert S.
Paudel, Liladhar
Paul, Matthew J.
Paull, Jeffrey
Paulmurugan, Ramasamy
Paulsen, Berit Smestad
Pavlidis, Ioannis
Pavón, Francisco Javier
Pawelec, B.
Pearce, A. Norrie
Pecht, Israel
Pecul, Magdalena
Peddabuddi, Gopal
Pediani, John
Pedraza-Chaverri, José
Pedro, Luis G.
Peisert, Heiko
Pelaez, José Walter
Pelicci, Pier G.
Pellei, Maura
Pellequer, Jean-luc
Pelletier, Jerry
Pelosi, Giorgio
Peltzer, M.
Pena Ruiz, T
Pěnčíková, Kristýna
Peng, Ai-Yun
Peng, Jin-Chyau
Peng, Jing
Peng, Junhua
Peng, Ling
Peng, Robert Y.
Peng, Wen-Huang
Peng, Xianghong
Peng, Xiaojun
Peng, Xinhua
Peng, Zhi-Yong
Peppel, Karsten
Pereda-Miranda, Rogelio
Peregrina, Jesus Manuel
Pereira, Letricia Barbosa
Pérez, Andy J.
Pérez, Silvia
Pérez-Álvarez, J.a.
Perez-Benito, Joaquin F.
Pérez-Correa, J. Ricardo
Perez-Costa, Emma
Pericàs, Miquel A.
Perjési, Pál
Perona, John J.
Perrin, Charles
Perrin, David
Perrow, Kara
Perry, Nigel
Perticone, Francesco
Pertsemlidis, Alexander
Pescitelli, Gennaro
Pestov, A.v.
Péter, Francisc
Peterka, František
Peters, Verena
Petretto, Giacomo L.
Petroni, Katia
Petzer, Jacobus P.
Peuchmaur, Marine
Pfaltz, Andreas
Pfeffer, Frederick M.
Phan, Manh-huong
Pi, Rongbiao
Piaz, Fabrizio Dal
Picariello, Gianluca
Pichert, Annelie
Picking, William D.
Pietras, Alexander
Pietro, Roberta Di
Pietruszka, Jörg
Pignatello, Joseph J
Pii, Youri
Pilc, Andrzej
Pillay, Viness
Piluzza, Giovannella
PINA, Fernando
Pina, João
Pinent, Montserrat
Pinheiro, Carla
Pinheiro, Maria
Pinho, Eva
Pinilla, Clemencia
Pintado, Manuela
Pinto, Diana C. G. A.
Piorkowska, Ewa
Piotrowska, Dorota G.
Piotrowska, Hanna
Pipelier, Muriel
Pirali, Tracey
Pirngruber, Gerhard D.
Pitchiaya, Sethuramasundaram
Pitsikalis, Marinos
Pitucha, Monika
Pitzurra, Lucia
Pizzala, Roberto
Plano, Daniel
Plastina, Pierluigi
Plech, Tomasz
Plourde, Guy L.
Pluen, Alain
Pócsi, István
Pocurull, Eva
Poczai, Péter
Poddel'sky, Andrey I.
Podolak, Irma
Poerschmann, Juergen
Poetsch, Micaela
Pogoń, Krystyna
Pogorelko, Gennady V.
Pohl, Pawel
Poinsot, Verena
Poleshchuk, Oleg
Poleszak, Ewa
Poletto, Matheus
Polson, Shawn W.
Polyak, Steven
Pontiki, Eleni
Popiołek, Łukasz
Poplawska, Magdalena
Popov, S.V.
Popovic, Boris
Poppe, László
Popsavin, Velimir
Porcheddu, Andrea
Porta, Francesca
Porter, John R.
Portilla Salinas, Jaime
Portillo, María Puy
Porzel, Andrea
Pose, Eva
Pöthig, Alexander
Potterat, Olivier
Poulas, Konstantinos
Poupaert, Jacques H.
Pour, Milan
Poyatos, Macrena
Pratsinis, Harris
Pratt, Stephen
Premkumar, Daniel R.
Prenner, Elmar
Prézeau, Laurent
Procopio, Jesús R.
Proescholdt, Martin
Proestos, Charalampos
Pronin, Alexander V.
Prosen, Helena
Protti, Stefano
Prousis, Kyriakos
Psarra, Anna-maria G.
Psomas, George
Pu, Xia
Pu, Xiaoping
Puglia, Carmelo
Punta, Carlo
Puntoriero, Fausto
Punzo, Francesco
Pusztaszeri, Marc
Pyrzynska, Krystyna
Pytkowska, Katarzyna
Qi, Xin
Qian, Bing-Jun
Qian, Hong
Qiao, Chunhua
Qin, Bo
Qin, Chuanguang
Qin, Hailin
Qin, Lei
Qin, Lu-Ping
Qin, Xuemei
Qiu, Feng
Qiu, Hongdeng
Qiu, Jianhui
Qiu, Ming-hua
Quadrelli, Paolo
Quan, Taihao
Quan, Zheshan
Quinn, Ronald
Quintard, Jean-Paul
Rablen, Paul R.
Racchi, M. L.
Racz, I.
Raczyńska, E. D.
Radi, Marco
Radominska-Pandya, Anna
Raederstorff, Daniel
Raffa, Vittoria
Raffaele, Frazzi
Raffaelli, Nadia
Ragaini, Fabio
Ragno, Gaetano
Ragno, Rino
Rai, Dilip K.
Raillard, Cécile
Rak, Janusz
Rakanovic-Todic, Maida
Rakitin, Oleg
Rakotondraibe, L.H.
Ralston, Katherine
Ramalhosa, Elsa
Ramesh, Rajagopal
Ramig, Keith
Ramos, Márcio Viana
Ramsay, Rona R.
Ramsh, S. M.
Rana, D.
Ranieri, Annamaria
Rao, P. N. Praveen
Rao, Zhiming
Rapisarda, Paolo
Rapposelli, Simona
Rasmussen, Seth C.
Rasulev, Bakhtiyor
Rau, Sven
Rauhut, Guntram
Rautio, Jarkko
Ravu, Ranga Rao
Rawel, H. M.
Rawlings, Neil D.
Rawls, Henry
Ray, Ratna
Reboul, Vincent
Reddy, M. V. Ramana
Redfors, Björn
Rehman, Junaid U.
Reible, Danny
Reich, Adam
Reigosa, Manuel J
Reiner, Martin
Reiser, Oliver
Reisman, David
Reisman, Scott A.
Remelli, Maurizio
Remião, Fernando
Ren, Gui-Xing
Ren, Jian-guo
Ren, S. P.
Renault, Hugues
Renčiuk, Daniel
Rendueles, Olaya
Renner, Ulrich
Reta, Mario
Revilla, Eugenio
Reyes, Fernando
Reynal, Anna
Reynisson, Jóhannes
Rho, Jung-Rae
Ribeiro, Bernardo Dias
Ricca, Ezio
Riccardi, Carlo
Ricci, Alfredo
Richomme, Pascal
Richter, Wito
Rico, Milagros
Riedmaier, Irmgard
Riela, Serena
Rigano, Daniela
Rijo, Patrícia
Rimoldi, Isabella
Rinaldi, A. C.
Rinaldi, Alessandra
Rinaudo, Marguerite
Rios, Yolanda
Riou, Laurent M.
Riu-Aumatell, Montserrat
Rivera Sánchez, Gildardo
Rivière, Céline
Rizzolo, A.
Rizzuti, Bruno
Roberge, Michel
Roberti, Marinella
Robina, Inmaculada
Robinson, Dominic J.
Rocamora, Mercè
Roca-Sanjuán, Daniel
Rocheleau, Jonathan V.
Rodilla, Jesus M.
Rodiño, Paula
Rodolfo, Carlo
Rodrigues, Ana Lúcia S.
Rodrigues, Rafael Costa
Rodrigues, S
Rodriguez, Laura
Rodríguez-Gonzalo, Encarnación
Rodriguez-Velamazan, Jose Alberto
Roes, Marilise Le
Roesler, R.
Rogers, Natasha
Rohn, Sascha
Roland, Sylvain
Roleira, Fernanda M.F.
Rolfe, Barbara E.
Rolfe, K. J.
Rolim Medeiros, Jand-venes
Rolle, Luca
Romagnoli, Romeo
Romanelli, Gustavo P.
Romero, M. Angeles
Romero-Castro, Aurelio
Rondeau, Philippe
Rong, Jianhui
Ros, M. Blanca
Rosa, Luis
Rosati, Ornelio
Rosato, Antonio
Roseiro, Luísa B.
Rosenberger, Thad A.
Roskoski, Robert
Ross, Carolyn F.
Ross, Samir
Rosselli, Sergio
Rossi, Elisabetta
Rossius, Vassilios
Rothenberg, Gadi
Rothstein, Jonathan P.
Roubelakis, Emmanuel
Rouseff, Russell L.
Routray, Winny
Rowley, Christopher
Rozen, Shlomo
Ruan, Han-Li
Rubert, Josep
Rubis, Blazej
Rudolf, Emil
Rudzitis-Auth, Jeannette
Ruggerone, Paolo
Ruijter, Dr E.
Ruijter, Eelco
Ruiz, José
Ruiz-Azuara, Lena
Russo, Gian Luigi
Russo, Mario Vincenzo
Russowski, Dennis
Russu, Wade A.
Ruud, Kenneth
Ryan, Geraldine D.
Ryu, Jae-Ha
Ryu, Shi Yong
Saaby, Lasse
Saavedra, Lucila
Sabila, Paul Sali
Sabio, Guadalupe
Sadekar, Shraddha
Safe, Stephen
Sagredo, Onintza
Saha, Achinto
Saito, Shinichi
Saito, Takao
Sakai, Ryuichi
Sakakibara, Hiroyuki
Sakamoto, Naoya
Sakwe, Amos
Salas, Cristián
Salas, Juan M.
Salatino, Antonio
Saldívar-Guerra, Enrique
Saleh, Mahmoud A.
Salgado, Josefa
Salgueiro, Ligia
Salinas, Rosario
Saliu, Francesco
Salomon, Claudio J
Salter, Russell
Salvalaglio, Matteo
Samaroo, Diana
Šamec, Dunja
Samir, Bondock
Samson, Andre L.
Samuelsen, Anne Berit C.
Sánchez, Gerardo
Sánchez-Campos, Sonia
Sánchez-Fidalgo, Susana
Sanchez-Moreiras, Adela
Sánchez-Moreno, Concepción
Sánchez-Palomo, Eva
Sanchez-Sanz, Goar
Sanchiz, Joaquin
Sandwick, Roger
Sang, Shengming
Sanjust, Enrico
Sanny, Charles G.
Sano, Shigeki
Santilio, A.
Santillan, Rosa
Santini, Carlo
Santini, Daniele
Santos, Maria M. M.
Santos, Sónia A. O.
Sanz, Maria
Sanzani, Simona Marianna
Sârbu, Costel
Sassi, Paola
Sasson, Yoel
Sato, F.
Sato, Kenji
Sato, Yuichiro
Saturnino, Carmela
Sautreau, Asmita
Sava, Gianni
Savić, Snežana
Savoie, Jean-Michel
Saxena, Sunil
Scarso, Alessandro
Scendo, Mieczyslaw
Schapiro, Igor
Scheerens, Joseph C
Scheiner, Steve
Schepetkin, IA
Schiaffino, M. Romina
Schinzer, Dieter
Schiraldi, Chiari
Schirawski, Jan
Schirrmacher, Ralf
Schlaad, Helmut
Schlierf, Michael
Schmeda-Hirschmann, Guillermo
Schmelz, Eric A.
Schmid, Peter
Schmidt, Heinar
Schmidt, Jürgen
Schmidt, Marie
Schmidt, Thomas J.
Schmitzer, Andreea R.
Schneider, Bernd
Schneider, Gyula
Schnermann, Martin
Schnitzler, Egon
Schols, Dominique
Scholz, Bettina
Schottenberger, Herwig
Schubert, Mario
Schulz, Benjamin L.
Schulz, R.
Schulze, Benjamin
Schwalbe, Harald
Schwan, Adrian L.
Schwartz, Steven D.
Schwarz, Quenten
Schweizer, Frank
Scilimati, Antonio
Scott, Jason
Scotti, Marcus
Scozzafava, Andrea
Scriba, Gerhard
Seca, Ana
Sedlak, Milos
Seehase, Sophie
Seela, Frank
Seely, Katerin
Seidel, Véronique
Seidler, Daniela G.
Seifert, Karlheinz
Sekhar, J. A.
Selbo, PÅl K.
Selicharová, Irena
Selvaggini, Roberto
Semenov, Victor
Sempere, Lorenzo F.
Senanayake, Thulani H.
Senba, Masachika
Senchina, David S.
Sendker, Jandirk
Seneviratne, Chaminda J.
Seong, Yeon Hee
Serpersu, Engin H.
Serralheiro, Maria Luísa M.
Serrano, María
Setaka, Wataru
Sethi, Gautam
Settanni, L.
Setzer, William
Sevilla, María-Ángeles
Sevilla, Michael
Seyedi, Neda
Sforcin, José Maurício
Shah, Dilip
Shah, Rashmi R.
Shaikh, Mohammed Abid
Shalaby, T.
Shalliker, R.A.
Shan, Wei-Guang
Shao, Yawei
Sharma, Amar Deep
Sharma, Arun K
Sharma, Virender K.
Shaw, Arthur Y.
She, Zhigang
Sheldon, Roger
Shen, Haoyu
Shen, Jianfu
Shen, Jiangang
Shen, Jinfeng
Shen, Qi
Shen, Qiang
Shen, Yingjia
Shen, Yue-Mao
Shenderovich, Ilya G.
Sheng, Baiyang
Sherwin, Catherine M. T.
Sheu, Jyh-Horng
Shi, Daqing
Shi, Dayong
Shi, Donglu
Shi, Jie-hua
Shi, Weiming
Shi, Xiaodong
Shi, Yongquan
Shibahara, Fumitoshi
Shibata, Norio
Shigemori, Hideyuki
Shih, Mei-Hsiu
Shimada, Tsutomu
Shimizu, Kuniyoshi
Shimizu, Linda
Shimoda, Hiroshi
Shin, Hyung Shik
Shin, Injae
Shin, Seokmin
Shin, Sung Chul
Shipley, Paul
Shirahata, Takashi
Shmatov, M. L.
Shoyama, Yukihiro
Shukla, Shruti
Siddiqui, Rafat
Siewert, Bianka
Sikora, Ewa
Silaghi-Dumitrescu, Radu
Sillanpaa, Mika
Silva, Ana Rosa
Silva, Fátima Regina Mena Barreto
Silva, J. P. Sousa e
Silva, Luís R.
Silva, María Fernanda
Silva, Sandra D.
Silván, Jose M.
Silver, Lynn
Silvério, Flaviano Oliveira
Silvestri, Elena
Simal-Gándara, Jesús
Simirgiotis, Mario
Simmler, Charlotte
Simões, Maria Fátima
Simon, Hans-uwe
Simoneau, Philippe
Simonsen, Henrik
Simpson, Bradley S.
Sinclair, Andrew
Sindler-Kulyk, Marija
Singer, Robert D.
Singh, I.
Singh, Kumud K.
Singh, Vijay K.
Singleton, Daniel A
Siniscalco, Dario
Siren, Heli
Sirover, Michael
Sissi, Claudia
Siu, Parco M.
Sivamani, Raja K
Siwek, Agata
Skalicka-Woźniak, Krystyna
Skoda-Földes, Rita
Sköld, Christian
Skrobik, Yoanna
Slavov, Anton
Sławiński, Jarosław
Sliva, Daniel
Sloan, Kenneth B.
Słoczyńska, Karolina
Slominski, Andrzej
Slusarenko, Alan J.
Smagghe, Guy
Šmejkal, Karel
Smietana, Michael
Smith, Graham
Smith, Keith
Smith, Martyn T.
Smith, Paul A.
Smith, Robert E.
Smoglia, James M.
Smyth, Thomas J.
Snow, Nicholas
Snyder, James P.
Sobarzo-Sánchez, Eduardo
Sobczak, Marcin
Sobotik, Pavel
Södergård, Anders
Soengas, Raquel
Sokovic, Marina
Solans-Monfort, Xavier
Soldan, Samantha
Solioz, Marc
Soll, Dieter
Solla, Humberto
Somoza, álvaro
Song, Fengrui
Song, Yang-heon
Song, Zhihong
Sopade, Peter A.
Sorci, Leonardo
Soreq, Hermona
Soriano-García, Manuel
Sorice, Maurizio
Sorokin, A.b.
Sorrenti, Valeria
Sosa, Alfonso T. García
Sosa, Silvio
Sosnovskikh, V. Ya.
Sosnowska, Anita
Soto, Carmen
Soubhye, Jalal
Sousa, Hugo
Sousa, M.C.
Sousa, Sérgio Filipe
Souza-Fagundes, Elaine M.
Sovage, Imre
Sowers, Lawrence C.
Sozzi, Gabriella
Spadavecchia, Jolanda
Spampinato, Santi
Sparagano, Olivier AE
Spatafora, Carmela
Speeckaert, Marijn
Spendier, Kathrin
Spitalsky, Zdenko
Spiteller, Michael
Spittau, Björn
Sporn, Michael B.
Spring, David
Spring, Otmar
Squina, F.M.
Srivenugopal, Kalkunte
Stadler, Marc
Staerk, Dan
Staiger, Christopher J.
Stajic, Mirjana
Stamatatos, Theocharis C.
Stamatis, Haralambos
Stanković, Milan S.
Stanley, Joanna L.
Stanovnik, Branko
Stavber, Stojan
Stec, Jozef
Stefanovic, Branko
Steindel, Mário
Steinmann, Eike
Stephanidou-Stephanatou, Julia
Stephen Cho Wing, Sze
Stępkowski, Tomasz M.
Stevanato, Roberto
Steven, Sebastian
Stevenson, Bradley
Stevenson, Julian
Stewart, Michael
Stiehler, Maik
Stiff-Roberts, Adrienne D
Stochmal, Anna
Stoika, R.S.
Stojakowska, Anna
Stojanovic, Ivana
Stone, Koni
Stone, Michael
Stoppacher, Norbert
Stortz, Carlos A.
Strand, Daniel
Stranger, Rob
Streit, Wolfgang R.
Strekowski, Lucjan
Struga, Marta
Su, Nan-Wei
Su, Shu-Jem
Su, Wen-Da
Su, Zhushang
Su, Zi-Ren
Suárez, Juan
Sugahara, Takuya
Sugai, Takeshi
Sugawara, Akira
Sugiura, Yoshimasa
Sugumaran, Manickam
Suh, Hyung Joo
Suib, Steven L.
Sukumar, N.
Sulová, Zdenka
Sülzmann, Holger
Sumarah, Mark
Sun, Bai-Wang
Sun, Changhao
Sun, Cheng
Sun, Grace
Sun, Hongzhe
Sun, Hui
Sun, Runcang
Sun, Yongxue
Sung, Jung-Suk
Sung, Ping-Jyun
Sun-Waterhouse, Dongxiao
Süss-Fink, Georg
Sussman, Mark A.
Sutherland, Hamish S.
Sutherland, J.B.
Sutter, E.M.M.
Suzuki, Hideyuki
Suzuki, Hiromu
Suzuki, Masahito
Suzuki, Tadashi
Suzuki, Takafumi
Suzuki, Takahiro
Suzuki, Takanori
Suzuki, Takuya
Svensson, Sara
Syed, Moinuddin
Szczepanik, Beata
Szekeres, Thomas
Szopa, Agnieszka
Sztanke, Małgorzata
Tabata, Keiichi
Tacconelli, Stefania
Tachikawa, Masanori
Tacke, Matthias
Taddei, Maurizio
Tadic, Vanja M.
Taghibiglou, Changiz
Taglialatela-Scafati, Orazio
Tagliazucchi, Davide
Tago, Megumi
Tai, Wanyi
Taillefumier, C.
Taira, Junsei
Takada, Yoshikazu
Takagi, Masatoshi
Takano, Toshiyuki
Takao, Ken-ichi
Takasu, Kiyosei
Takayanagi, Toshiyuki
Takeda, Makio
Takeda, Youhei
Takemori, Hiroshi
Takemura, Genzou
Taki, Masumi
Takii, Takemasa
Talbot, Nicholas J.
Talele, Tanaji T
Tam, Kam Chiu
Tamariz, Joaquín
Tamiaki, Hitoshi
Tan, Chin Ping
Tan, Dun-Xian
Tan, Jian-wen
Tanaka, Akane
Tanaka, Hitoshi
Tanaka, Shunsuke
Tang, Dingqin
Tang, Feng-Yao
Tang, Tao
Tang, Wei
Tang, Xing
Tang, Yiqun
Tang, Yuping
Tanga, Mary
Taniguhci, Takaaki
Tantillo, Dean
Taran, Frédéric
Tarozzi, Andrea
Tarr, Matthew A.
Tateishi-Karimata, Hisae
Tattini, Massimiliano
Taurin, Sebastien
Taveira, Marcos
Taylor, Michelle
Taylor, Peter
Taylor, T. Matthew
Teixeira, Cátia
Teixeira, Róbson
Teixidor, Francesc
Teng, Yuanwen
Teppema, Luc J.
Terekhova, Irina V.
Tesarová, Eva
Teschke, Rolf
Tesmer, John J. G.
Thadani, Udho
Thakur, Mayank
Thèvenod, Frank
Thews, Oliver
Thirunavukkarasu, Mahesh
Thomas, Helen E.
Thomas, Raymond H.
Thompson, Paul R.
Thomson, Leonor
Thoo-Lin, Paul Kong
Thordarson, Pall
Tian, Ping
Timopheev, A.A.
Timoshenko, Alexander V.
Timperio, Anna Maria
Timsit, Youri
Titanyuk, Igor D.
Tobacman, Joanne K.
Tocchetti, Carlo G.
Todorovic, Tamara
Toegel, Stefan
Toida, Toshihiko
Tokuda, Gaku
Tokuda, Harukuni
Toma, Henrique E.
Tomečková, Vladimíra
Tommonaro, Giuseppina
Tonelli, Alan E.
Tong, Lingying
Tonnabel, Jeanne
Toohey, John I.
Topham, Paul D.
Topuzova-Hristova, Tanya
Tori, Motoo
Toropov, Andrey A
Torras, J.
Torre, Maria
Torre, Maria Luisa
Torreggiani, Armida
Torrens, Francisco
Toyomasu, Tomonobu
Toyooka, Naoki
Trabace, Luigia
Trabbic, Christopher
Tramontini, Sara
Tran, Clara T. H.
Trindade, Alexandre
Trindade, Reginaldo Almeida da
Trivedi, Prabodh Kumar
Trougakos, Ioannis
Trouillas, Patrick
Trudell, Mark
Tsai, C.Y.
Tsai, Cheng-Fang
Tsai, Shau-Wei
Tselios, Theodore
Tseng, Chao-neng
Tsenkova, Roumiana
Tsim, Karl W.K
Tsipis, Athanassios C.
Tsubaki, Shuntaro
Tsuge, Takeharu
Tsukamoto, Koji
Tsukiyama-Koharaemail, Kyoko
Tsunemasa, Noritaka
Tsutsui, Shigeyuki
Tu, Pengfei
Tuberoso, Carlo
Tulla-Puche, Judit
Tuñón, Iñaki
Turck, Natacha
Turel, Iztok
Turks, Māris
Turło, Jadwiga
Tuttolomondo, M.E.
Tyler, Betty
Tzeli, Demeter
Tzia, C.
Ubiali, Daniela
Uchi, Hiroshi
Uchida, Yoshikazu
Uddin, Md. Jashim
Uddin, Zakir
Ueda, Mitsuhiro
Ueda, Natsuo
Ueda-Nakamura, Tânia
Ueno, Makoto
Uhrin, Dusan
Uivarosi, Valentina
Ukic, Sime
Ulanski, Piotr
Ulber, Roland
Ulrih, Nataša Poklar
Uludağ, Hasan
Unkefer, Clifford J.
Upadhyay, Atul
Urbano-Cuadrado, Manuel
Urbieta, María Tabernero
Uriarte, Eugenio
Usami, Yoshihide
Uto, Yoshikazu
Uyama, Hiroshi
Vacek, Jan
Vale, Nuno
Valentao, Patrícia
Valero, Francisco
Valero, M.
Valgimigli, L.
Vallés, Cristina
Valmikanathan, O.P.
Van Aerschot, Arthur
Van Calenbergh, Serge
Van De Venter, Maryna
Van den Ende, Wim
van der Meijden, Maarten
Van Der Veken, Pieter
Van Griensven, Leo J.L.D
Van Hook, M.J.
Van Kampen, Jackalina M.
van Thor, Jasper J.
Vanden Eynde, Jean Jacques
Vanderlei de Souza, Maria de Fátima
Vankempen, Leon
Vankova, Radomira
Váradi, Györgyi
Varela-Ramirez, Armando
Varese, Giovanna Cristina
Vargas-Bello-Pérez, E.
Vargas-Rodriguez, Yalma L.
Varrot, Annabelle
Varvounis, George
Vasconcelos, Helena
Vasilakoglou, Ioannis
Vasile, Bogdan Stefan
Vautard, Frederic
Vávrová, Kateřina
Vaz, Deisi Altmajer
Vaziri, Nosratola D.
Vázquez-Flota, Felipe
Vecchione, Carmine
Vegvari, Akos
Veiga-Junior, Valdir
Velasco, Pablo
Velez, Zélia
Veneziani, Rodrigo
Venkataraman, Shrinivas
Venturella, Giuseppe
Venukadasula, Phanindra
Veres, Balazs
Verhelst, Steven
Very, Thibaut
Vesuna, Farhad
Vetter, Walter
Vetvicka, Vaclav
Vidanarachchi, Janak K.
Vidossich, Pietro
Vieira, Mónica
Vigier, Karine
Vijver, Pieter Van de
Vila, José Manuel
Vilar, Vítor J.P.
Vilela, Alice
Villani, Claudio
Villegas, Alejandro Madrid
Virkel, Guillermo
Virlogeux-Payant, Isabelle
Visioli, Francesco
Vistnes, Maria
Viuda-Martos, Manuel
Vivas-Reyes, Ricardo
Vo-Dinh, Tuan
Vomiero, Alberto
Von Knethen, Andreas
Vorsa, Nicholi
Voss, Jürgen
Voutquenne, Laurence
Vrchotová, Naděžda
Vuong, Quan V.
Wagner, Caroline
Wagner, Gergard
Wahl, M. A.
Waksmundzka-Hajnos, Monika
Walker, Anne-sophie
Wallis, Russell
Walorczyk, Stanisław
Wan, David CC
Wan, Jian-bo
Wanderley, Almir G.
Wang, Chi Chiu
Wang, Ching-Chiung
Wang, Chin-Kun
Wang, Cunde
Wang, Deyun
Wang, Fei
Wang, Guangshun
Wang, Hexiang
Wang, Hongbing
Wang, Hongjun
Wang, Horng-dar
Wang, Jeh-jeng
Wang, Jiadao
Wang, jianguo
Wang, Jianji
Wang, Jianping
Wang, Junzhi
Wang, Kai
Wang, Ke-Rang
Wang, Lai-xi
Wang, Minghua
Wang, Peng George
Wang, Qiangbin
Wang, Qun
Wang, Rui
Wang, Shaoyun
Wang, Sheng-Yang
Wang, Shun-Yi
Wang, Shusheng
Wang, Thomas T. Y.
Wang, Wei-hsien
Wang, Wei-Ning
Wang, Xia
Wang, Xiao-Bing
Wang, Xiaoying
Wang, Xiaoyong
Wang, Xinluan
Wang, Xuedong
Wang, Xumei
Wang, Yonghua
Wang, Youwei
Wang, Yuanpeng
Wang, Yue-Hu
Wang, Yuqi
Wang, Zhao-Yang
Wang, Zhimin
Wang, Zhiwen
Wang, Zhiyong
Wängler, Björn
Want, Elizabeth J.
Ward, Dale E.
Wardrop, Duncan J.
Warmuth, Ralf
Watanabe, Eiki
Watanabe, Kazuki
Watanabe, M
Watanabe, Naoki
Watanabe, Yasuharu
Waterschoot, Jasmien
Webb, Thomas
Webber, Ralf
Weber, Bernd
Weber, Heike
Weber-Ban, Eilika
Wei, Na
Wei, Xiao-Yi
Wei, Zhiyong
Weigand, Wolfgang
Weingart, Georg
Weiss, Clemens K.
Weiss, H. Markus
Welch, K. D.
Weldon, Sinead
Wells, Shawn
Wen, Kuo-Ching
Wenthold, Paul G
Wenzel, Anna G.
Werner, Maria
Werz, Daniel
Wesolowski, Marek
West, James D.
Westerlind, Ulrika
Westman, Gunnar
Weston, Leslie
Westwell, Andrew D.
Wherland, Scot
Whitlock, Christine
Whitman, Malcolm
Whyte, Claire
Wichapong, Kanin
Wicker, Louise
Wiczk, Wiesław
Wiczkowski, Wieslaw
Wieczorek, Piotr
Wielgomas, Bartosz
Wijkander, Jonny
Wilden, Jonathan
Williams, David M.
Williams, Noelle S.
Williams, Spencer
Williamson, Gary
Willmore, William G.
Wills, Martin
Wilson, James N.
Wilson, Lee D.
Winckler, Thomas
Windey, Karen
Wink, Michael
Winnicka, Katarzyna
Winssinger, Nicolas
Winterhalter, Mathias
Wise, Steven G
Wishart, Jane
Wiskur, Sheryl L.
Wittmann, Jürgen
Wittrant, Yohann
Wnuk, Stan
Woelwer-Rieck, Ursula
Wójciak-Kosior, Magdalena
Wojdylo, Aneta
Wojnárovits, Laszlo
Wolan, Dennis W.
Wolska, Krystyna I.
Wong, C K
Wong, Danny K. Y.
Wong, Fung Fuh
Wong, Jonathan Woon Chung
Wong, Ka-Leung
Wong, Kenneth K. Y.
Wong, Man-Sau
Woo, Mi Hee
Wood, W. Gibson
Workentin, Mark S.
Wormley, Jr., Floyd L.
Wöstemeyer, Johannes
Wright, Clyde J.
Wright, Dennis
Wright, Louwrance
Wrolstad, Ronald E.
Wu, Bin
Wu, Chieh-Hsi
Wu, Chin-Chung
Wu, Chi-Rei
Wu, Chung-Yi
Wu, Ho-Shing
Wu, Huayue
Wu, Jianyong
Wu, Jyh-Horng
Wu, Li-chen
Wu, Ming-chang
Wu, Qi
Wu, Ruilian
Wu, Shihua
Wu, Tao
Wu, Tzu-Hua
Wu, Xiao-Feng
Wu, Yisheng
Wu, Yu-Tse
Wu, Yuyang
Wu, Zhaofei
Wube, Abraham Abebe
Wuest, Frank
Wujec, Monika
Wybraniec, Slawomir
Wysocki, Laura
Wyzgoski, Faith J.
Xavier, Nuno Manuel
Xia, Wenshui
Xian, Ming
Xiang, Lan
Xiao, Dong-Guang
Xiao, Sulong
Xiao, Yongsheng
Xiao, Zhousheng
Xie, Hua
Xie, Qian
Xie, Xiaoliang Sunney
Xie, Yong-Mei
Xin, Zhihong
Xiong, Bing
Xiong, Gaofeng
Yan, Xiongo
Xu, Baoshan
Xu, Changjie
Xu, Feng
Xu, Guobao
Xu, Peng-Fei
Xu, Qiong-ming
Xu, Shichao
Xu, Weiwei
Xu, Wen-Fang
Xu, Xiao-Jun
Xu, Xudong
Xu, Xueming
Xu, Yan
Xu, Yi-Jun
Xu, Zheng-Hong
Xu, Zhimin
Xue, Ming
Yaegaki, Ken
Yagi, Akira
Yagüe, Ernesto
Yahara, Shoji
Yamada, Shinji
Yamada, Shuhei
Yamagishi, Nobuyuki
Yamaki, Koji
Yamamoto, Hisashi
Yamanaka, Masamichi
Yamasaki, Sho
Yamashita, Fumiyoshi
Yamato, Takehiko
Yamauchi, Satoshi
Yan, Chao-Guo
Yan, Fei
Yan, Jizhong
Yan, Rulong
Yan, Xiufeng
Yan, Yunjun
Yan, Yun-jun
Yanez, Claudia
Yang, Chao-Hsun
Yang, Chunping
Yang, Depo
Yang, Ding-Yah
Yang, Guang-Fu
Yang, Haw
Yang, Hongjun
Yang, Hyun Ok
Yang, Li
Yang, Ling
Yang, Liqun
Yang, Liuqing
Yang, Paul
Yang, Qing
Yang, Shun-Fa
Yang, Song
Yang, Wei
Yang, Wei-hsiung
Yang, Wen-bin
Yang, Xian-wen
Yang, Xiuwei
Yang, Yong-hua
Yang, Yu-Chiao
Yang, Yu-Rong
Yang, Yushe
Yang, Z.-Q.
Yang, Zhijun
Yao, Guangmin
Yao, Jianxiu
Yasuda, Midori
Yasuhiro, SHINKAI
Ye, Jia-Hai
Ye, Jiandong
Ye, Min
Ye, Xingqian
Yeh, Jui-Ming
Yen, Gow-Chin
Yepuri, Nageshwar R.
Yerabolu, Jayasudhan Reddy
Yeung, Ying-yeung
Yi, Hankuil
Yi, Jianhua
Yi, Tae-hoo
Yi, Tao
Yi, Zhengping
Yiap, Beow Chin
Zhao, Yilei
Yim, Cheng-Bin
Yin, Hong
Yin, Mei-Chin
Yin, Ruohe
Yin, Shou-Wei
Yin, Shuang-Feng
Ying, Liu
Yokomatsu, Tsutomu
Yoneyama, Koichi
Yoo, Byoungseung
Yoo, Kyung Ho
Yoo, Yeong-Min
Yoo, Yung-Choon
Yoon, Do-Young
Yoon, Kyung Byung
Yoon, Moon-Young
Yoon, Seung Chul
Yordanov, Georgi
You, Liang
Yu, Yan-Ying
Yu, Guipeng
Yu, Li
Yu, Longjiang
Yu, Rongmin
Yu, Shi-shan
Yu, Shuhui
Yu, Ting
Yu, Xiaoqi
Yu, Ying
Yu, Yinghua
Yu, Yongping
Yu, Yuan-Hsiang
Yu, Yun-Qiu
Yu, Zhengkun
Yuan, Gu
Yue, Patrick Ying Kit
Yue, Yong-De
Yuliarti, Oni
Yun, Hyo-In
Yun, Junxian
Yun, Young Sook
Zabaras, Dimitrios
Zaferanloo, Bita
Zaikov, Gennadi
Zaleska, Adriana Zaleska
Zampieri, Daniele
Zapotoczna, Marta
Zarejousheghani, Mashaalah
Zarrelli, Armando
Zavala, Jorge A.
Zavodnik, Ilya
Zaware, Nilesh
Zawilska, Jolanta B.
Zehl, Martin
Zeiner, Tim
Zelisko, Paul M.
Zemkova, H.
Zeng, Guang Ming
Zeng, H.
Zeng, Runying
Zeng, Xiaoming
Zerbetto, Francesco
Zgoła-Grześkowiak, Agnieszka
Zhai, Haiyan
Zhang, Can
Zhang, Changhe
Zhang, Datong
Zhang, Dayi
Zhang, Dengsong
Zhang, De-Qiang
Zhang, Donglin
Zhang, Dong-ming
Zhang, Donna
Zhang, Guolin
Zhang, Guoqing
Zhang, Haifei
Zhang, Han-ting
Zhang, Hong-jie
Zhang, Jie
Zhang, Jinbiao
Zhang, Lin
Zhang, Min
Zhang, Ming-Rong
Zhang, Ning
Zhang, Qian
Zhang, Qingwen
Zhang, Shicheng
Zhang, Shijun
Zhang, Songping
Zhang, Tong
Zhang, X. Q.
Zhang, Xiang
Zhang, Xiaoming
Zhang, Xiaoying
Zhang, Xiaoyue
Zhang, Xiquan
Zhang, Xiuli
Zhang, Xiurong
Zhang, Yan
Zhang, Ying
Zhang, Yingrui
Zhang, Yong
Zhang, Yonghui
Zhang, Zhanhui
Zhang, Zhenwen
Zhao, Bao-xiang
Zhao, Bin
Zhao, Boxin
Zhao, Gui-sen
Zhao, Guohua
Zhao, Guoqiang
Zhao, Hong
Zhao, Hua
Zhao, Jian
Zhao, Jin-Hao
Zhao, Qinshi
Zhao, Wei
Zhao, Wei-Guang
Zhao, Weirong
Zhao, Xin-Qing
Zhao, Youxin
Zhao, Yuan
Zhao, Zhenjiang
Zheng, Jian-Bin
Zheng, Yu-Guo
Zhou, Zhongjun
Zhou, Guang-Biao
Zhou, Guang-Xiong
Zhou, Huchen
Zhou, Jia
Zhou, Tao
Zhou, Wei-Qing
Zhou, Xiaolong
Zhou, Yifa
Zhou, Zheng-Hong
Zhou, Zhi-Ming
Zhou, Zhongjun
Zhu, Changfu
Zhu, Fan
Zhu, Gangguo
Zhu, Guo-Yuan
Zhu, Jun
Zhu, Xiaofeng
Zhu, Xuan
Zhu, Ying
Zhu, Yinghuai
Zhu, Yulei
Zielske, Steven P.
Zitko, Jan
Złotek, Urszula
Zografos, Alandros L.
Zollo, Massimo
Zong, Xuxiao
Zorc, Branka
Zou, Zhongmei
Zoumpoulakis, Panagiotis
Zovko Končić, Marijana
Zrnčić, Mirta
Zughaier, Susu M.
Zuk, Magdalena
Zuo, Li
Zupkó, István
Zvyagi, Andrei V.
Zygadlo, Julio A.

